# Suppression of HBV replication by the expression of nickase- and nuclease dead-Cas9

**DOI:** 10.1038/s41598-017-05905-w

**Published:** 2017-07-21

**Authors:** Takeshi Kurihara, Takasuke Fukuhara, Chikako Ono, Satomi Yamamoto, Kentaro Uemura, Toru Okamoto, Masaya Sugiyama, Daisuke Motooka, Shota Nakamura, Masato Ikawa, Masashi Mizokami, Yoshihiko Maehara, Yoshiharu Matsuura

**Affiliations:** 1 Department of Molecular Virology, Research Institute for Microbial Diseases, Osaka University, Osaka, Japan; 2 Graduate School of Medicine, Department of Surgery and Science, Kyushu University, Fukuoka, Japan; 3 The Research Center for Hepatitis and Immunology, National Center for Global Health and Medicine, Ichikawa, Japan; 4 Department of Infection Metagenomics, Research Institute for Microbial Diseases, Osaka University, Osaka, Japan; 5 Center for Genetic Analysis of Biological Responses, Research Institute for Microbial Diseases, Osaka University, Osaka, Japan; 6 Present Address: Laboratory of Veterinary Microbiology, School of Veterinary Medicine, Kitasato University, Higashi 23-35-1, Towada, Aomori 034-8628 Japan

## Abstract

Complete removal of hepatitis B virus (HBV) DNA from nuclei is difficult by the current therapies. Recent reports have shown that a novel genome-editing tool using Cas9 with a single-guide RNA (sgRNA) system can cleave the HBV genome *in vitro* and *in vivo*. However, induction of a double-strand break (DSB) on the targeted genome by Cas9 risks undesirable off-target cleavage on the host genome. Nickase-Cas9 cleaves a single strand of DNA, and thereby two sgRNAs are required for inducing DSBs. To avoid Cas9-induced off-target mutagenesis, we examined the effects of the expressions of nickase-Cas9 and nuclease dead Cas9 (d-Cas9) with sgRNAs on HBV replication. The expression of nickase-Cas9 with a pair of sgRNAs cleaved the target HBV genome and suppressed the viral-protein expression and HBV replication *in vitro*. Moreover, nickase-Cas9 with the sgRNA pair cleaved the targeted HBV genome in mouse liver. Interestingly, d-Cas9 expression with the sgRNAs also suppressed HBV replication *in vitro* without cleaving the HBV genome. These results suggest the possible use of nickase-Cas9 and d-Cas9 with a pair of sgRNAs for eliminating HBV DNA from the livers of chronic hepatitis B patients with low risk of undesirable off-target mutation on the host genome.

## Introduction

Hepatitis B virus (HBV) infects over 250 million people worldwide^1^. Chronic infection with HBV increases the risk for deadly complications, including cirrhosis and hepatocellular carcinoma, resulting in 600,000 deaths per year worldwide. HBV is a member of the *Hepadnaviridae* family, and its life cycle involves both DNA and RNA. The HBV genome exists in nuclei as a 3.2-kb double-stranded circular DNA called covalently closed circular DNA (cccDNA)^2^. The cccDNA is a key component in the HBV life cycle, since it is the template for all viral RNAs including pregenomic RNA (pgRNA) and mRNAs, and thus contributes to the preservation of HBV persistence in infected cells^3^.

Currently, nucleoside and nucleotide analogs are efficient anti-HBV therapeutics for inhibiting the reverse transcription of HBV genomes, but these analogs cannot completely eliminate HBV from infected cells due to the preservation of cccDNA in nuclei. Therefore, long-term treatment is required and sometimes leads to concomitant resistance^4^. Although interferon-alpha (IFN-α) treatment can clear HBV DNA in limited patients, high-dose or long-term treatment with IFN-α cannot be tolerated due to its side effects^5^. The complete removal of HBV DNA from infected cells is thus difficult to achieve with the currently available therapeutics, and overcoming this challenge is one of the major goals of HBV research. Recent investigations have shown that a novel gene-editing tool using the RNA-guided DNA endonuclease Cas9 (CRISPR-associated protein 9) with a single-guide RNA (sgRNA) system can cleave the HBV genome and suppress HBV infection^6–9^. In addition, gene-editing therapy including CRISPR (clustered regularly interspaced short palindromic repeats)/Cas9 technology for patients with HIV, leukemia or solid cancers has already been applied in clinical settings^10–13^.

As a gene-editing nuclease, Cas9 induces double-strand breaks (DSBs) on target DNA via recognition by sgRNAs^14–16^. Cas9-induced DSBs are repaired by non-homologous end joining (NHEJ) by which variable lengths of insertions or deletions at the site of the DSBs are generated. Although Cas9 expression with sgRNAs can conveniently induce mutagenesis on the target DNA, the risk of undesirable off-target mutagenesis on the host genome is high^17–19^. To increase the specificity on targeted DNA and reduce undesirable off-target mutagenesis on the host genome, a pair nicking strategy using nickase-Cas9 to inactivate either of the nuclease domains of RuvC and NHN was introduced^20, 21^.

Since nickase-Cas9 cleaves only a single strand of the target DNA, a pair of sgRNAs targeting both strands of DNA is required for the induction of DSBs on the target DNA, resulting in increased specificity and a reduction of the risk of undesirable off-target mutagenesis on the host genome. In the present study, we showed that nickase-Cas9 with a pair of sgRNAs targeting the HBV genome achieved effective cleavage and suppressed HBV replication. Interestingly, we also found that nuclease dead Cas9 (d-Cas9) expression with sgRNAs similarly suppressed HBV replication but without cleaving the HBV genome.

## Results

### Design and validation of sgRNAs targeting the HBV genome

**Figure 1:**
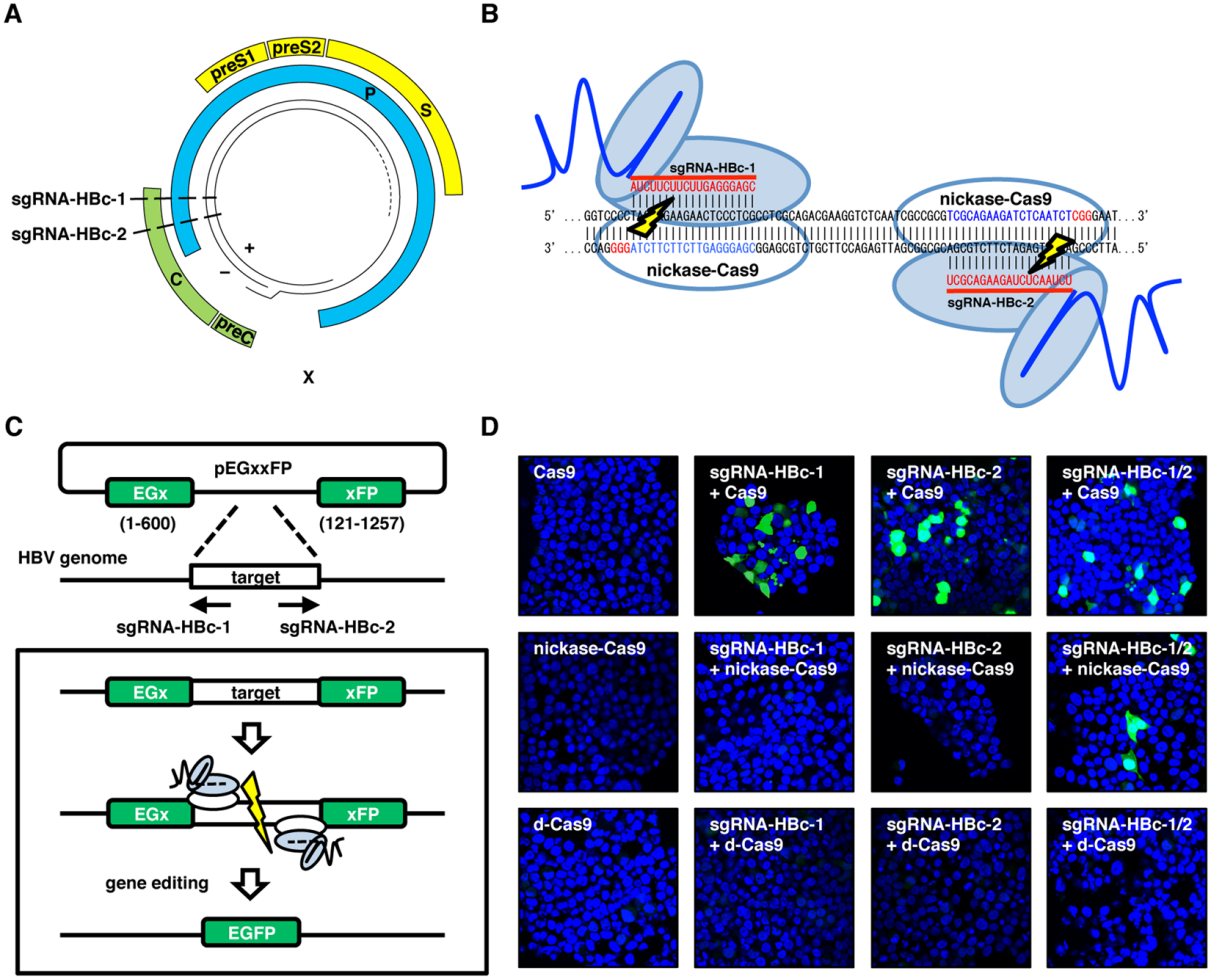
Cas9-based systems for gene editing and HBV-specific sgRNAs targeting sites for Cas9, nickase-Cas9 or d-Cas9. (**A**) Schematic diagram representation of the HBV genome. The four viral transcripts of the core are indicated: C, polymerase; P, surface; S and X, proteins. Regions targeted by sgRNAs are indicated. (**B**) The DNA sequences targeted by nickase-Cas9 with the pair sgRNA-HBc-1 and sgRNA-HBc-2 in the gene coding HBc protein. (**C**) Schematic of the EGxxFP assay performed to analyze the cleavage effects of Cas9, nickase-Cas9 and d-Cas9 expression with sgRNA-HBc-1 and sgRNA-HBc-2. A split EGxxFP plasmid is inserted with the targeted HBV sequence, and a recombination of EGFP is induced by DSBs on the targeted sequence. (**D**) Plasmids encoding Split EGxxFP possessing the HBc sequence, Cas9, nickase-Cas9, d-Cas9, and the sgRNAs were transfected into 293T cells. EGFP fluorescence was detected at 24 hr post-transduction.

Eight genotypes with sequence diversity have been identified in the HBV genome^22^. To target the HBV genome, we compared the sequences of several of these genotypes. Using the conserved sequences identified in the open reading frames encoding HBc protein (Fig. 1A), we designed two sgRNAs (which we named sgRNA-HBc-1 and sgRNA-HBc-2) that are complementary to 20 bp of both strands of HBV DNA adjacent to protospacer motif (PAM) sequences (Fig. 1B). To analyze the cleavability of nickase-Cas9 with a pair of sgRNAs targeting the HBc sequence, we constructed a split EGxxFP plasmid in which the target HBV sequence was inserted^23^.

The cleavage of each strand of the inserted HBV DNA leads to the recombination of EGFP, and thus the nuclease activity is visualized by the expression of EGFP (Fig. 1C). The expression of Cas9 with sgRNA-HBc-1 and/or sgRNA-HBc-2 in 293T cells induced the expression of EGFP, whereas both sgRNA-HBc-1 and sgRNA-HBc-2 were required for EGFP expression in the case of nickase-Cas9 expression. As we expected, the expression of d-Cas9 together with the sgRNAs could not induce the expression of EGFP (Fig. 1D).

Next, to examine the off-target mutagenesis on the host genome recognized by the sgRNAs, we selected the possible off-target sequences in the host genome by using CRISPRdirect^24^ and amplified the sequences in cells expressing the pair of the sgRNAs and the nucleases (Table 1). Our deep sequence analysis revealed that the frequency of mutations in the sites was below the detection limit of <0.1% (data not shown), suggesting that the co-expression of Cas9 with one sgRNA or nickase-Cas9 with a pair of sgRNAs is capable of inducing gene editing on targeted sequences. We then examined the effect of the co-expression of the nucleases with the sgRNAs on the replication of HBV.

**Table 1:** The target HBV sequences and the possible off-target sequences in the host genome.

Site name	Sequence
HBc-1	CCCTAGAAGAAGAACTCCCTCGC
Off-target #1–1	CC**T**TAGAAGAAGAACT**TG**C**C**CG**A**
Off-target #1–2	CCCTAGAAGAAGAACTCC**A**T**TTT**
Off-target #1–3	CCCTAGAAGAAGAACTC**A**C**CATT**
HBc-2	GTCGCAGAAGATCTCAATCTCGG
Off-target #2–1	**A**T**TCACT**AAGATCTCAATCTCGG
Off-target #2–2	**ACAAGG**GAAGATCTCAATCT**G**GG
Off-target #2–3	G**GAAG**A**C**AAGATCTCAATCT**G**GG

Underlining indicates the difference between the targeted HBV sequences and the candidate off-target sequences.

### The expression of Cas9 with both sgRNAs cleaved the target HBV genome and suppressed HBV replication

**Figure 2:**
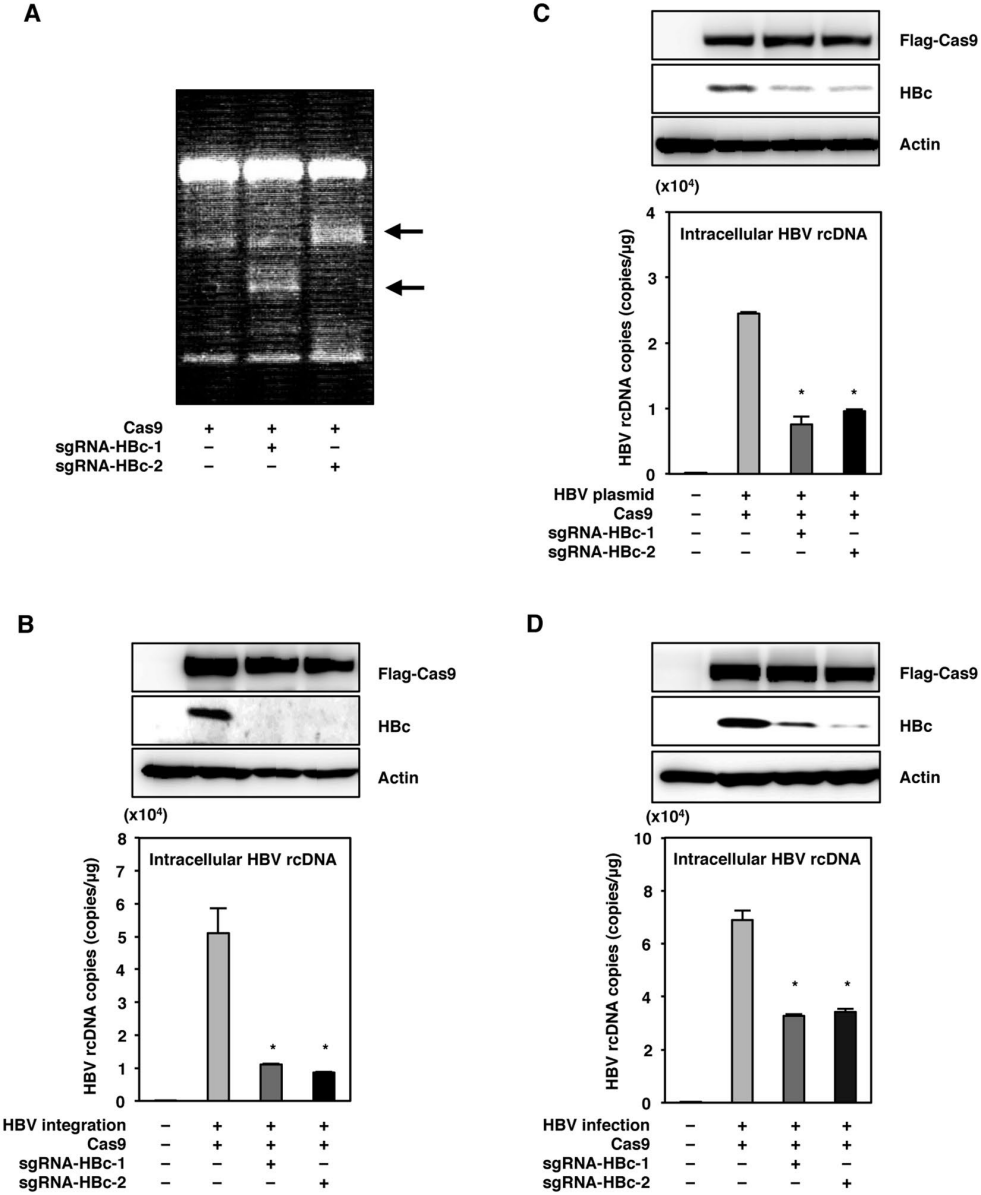
The expression of Cas9 with the sgRNAs can cleave the target HBV genome and suppress HBV replication. (**A**) Cas9 and either sgRNA-HBc-1 or sgRNA-HBc-2 were lentivirally transduced into HepG2.2.15.7 cells. Total DNA was collected at 5 days post-transduction, and mutations of the HBV genome in HepG2.2.15.7 cells were detected by a surveyor assay. The arrows indicated the surveyor digestion products. (**B**) HepG2.2.15.7 cells transduced with Cas9 and the sgRNAs were collected at 5 days post-transduction, and the expression of HBc was determined by immunoblotting. Intracellular HBV rcDNAs were extracted at 5 days post-transduction and quantified by qPCR. (**C**) Huh7 cells transduced with Cas9 and the sgRNAs were transfected with a plasmid containing 1.3-fold-overlength genome of HBV at 3 days post-transduction. Cell lysates were collected at 3 days post-transfection, and the expression of HBc was determined by immunoblotting. Intracellular HBV rcDNAs were extracted at 3 days post-transfection and quantified by qPCR. (**D**) HepG2-hNTCP-C4 cells expressing Cas9 and the sgRNAs by lentivirus vector were infected with HBV derived from the supernatants of HepAD38.7 cells at 3 days post-transduction. Cell lysates were collected at 10 days post-infection, and the expression of HBc protein was determined by immunoblotting. Intracellular HBV rcDNAs were extracted at 10 days post-infection and quantified by qPCR. *p < 0.01 vs. the results for control cells. (full-length gels are presented in Supplementary Figure).

To examine the effect of the expression of Cas9 together with sgRNA-HBc-1 and sgRNA-HBc-2 on the replication and cleavage of viral DNA of HBV, we used HepG2.2.15.7 cells (which integrate the HBV genomes and produce infectious particles) as a model of chronic infection^25^. To evaluate Cas9-mediated mutagenesis, we performed a surveyor assay for the detection of mutations by cleaving the mutated sequences in the target DNA. Introduction of mutations in the HBV genome was detected in HepG2.2.15.7 cells upon the expression of Cas9 with either of the sgRNAs by lentiviral vectors (Fig. 2A). The expression of Cas9 with sgRNA-HBc-1 or sgRNA-HBc-2 strongly suppressed the expression of HBc protein and the production of rcDNA in HepG2.2.15.7 cells (Fig. 2B).

We next examined the effect of Cas9 expression with the sgRNAs on the replication of HBV in Huh7 cells transfected with the plasmid containing 1.3-fold-overlength genome of HBV genotype C^26^. Cas9 expression with the sgRNAs suppressed the expression of HBc protein and the production of rcDNA in Huh7 cells (Fig. 2C). Additionally, we also examined the effect of Cas9 expression with the sgRNAs on HBV infection in HepG2-hNTCP-C4 cells, stably expressing human sodium taurocholate co-transporting polypeptide (hNTCP) as an HBV receptor^27, 28^. In the present experiments, HepG2-hNTCP-C4 cells expressing Cas9 and the sgRNAs by lentivirus vector were infected with HBV derived from the supernatants of HepAD38.7 cells. The expression of Cas9 with sgRNA-HBc-1 or sgRNA-HBc-2 suppressed the expression of HBc protein and the production of rcDNA in HepG2-hNTCP-C4 cells at 10 days post-infection with HBV (Fig. 2D). These results suggest that Cas9 expression with the sgRNAs can cleave the targeted HBV genome and suppress viral replication *in vitro*.

### The expression of nickase-Cas9 with one or both sgRNAs suppressed the replication of HBV

**Figure 3:**
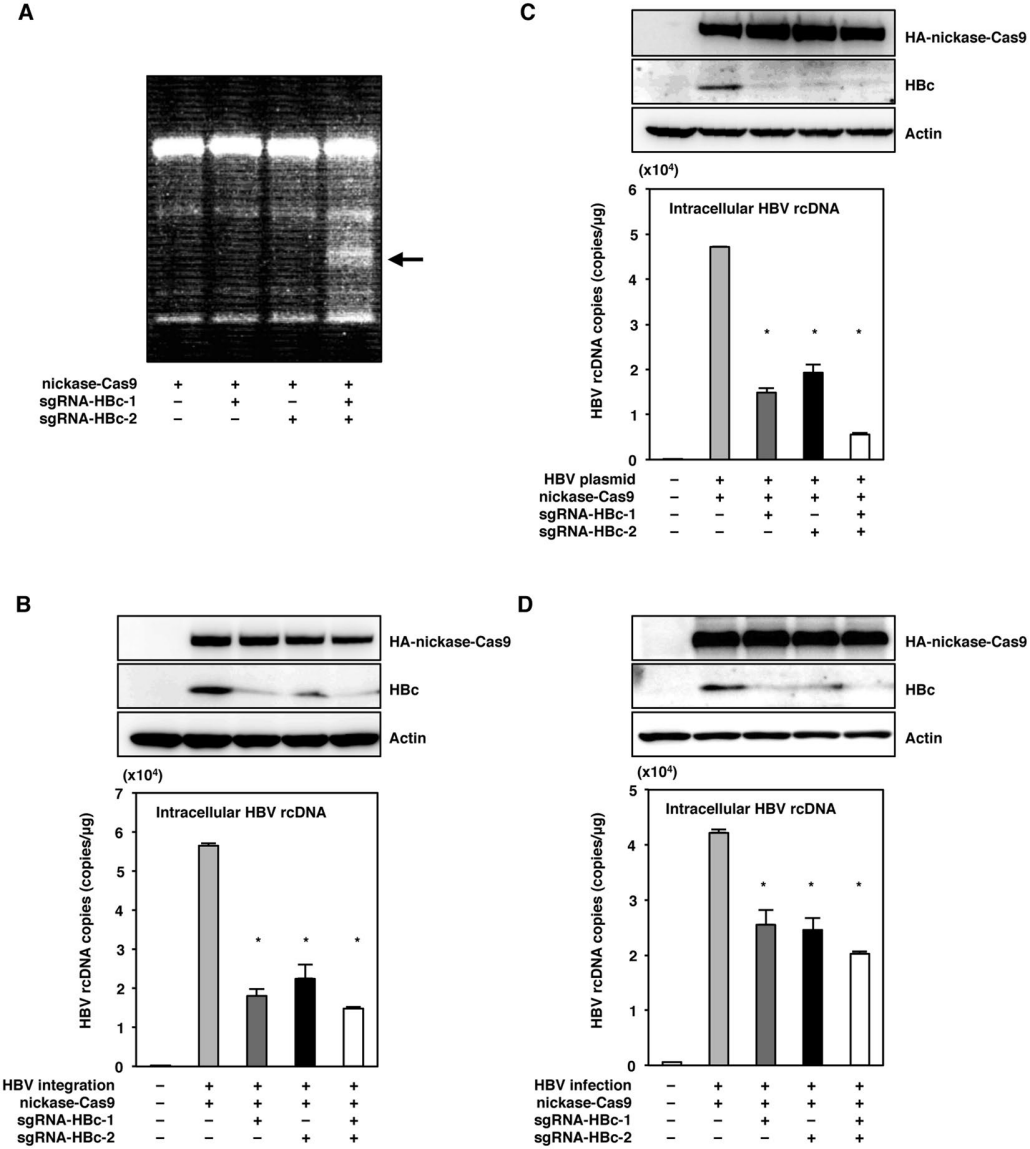
The expression of nickase-Cas9 with either or both of the sgRNAs suppressed the replication of HBV. (**A**) Nickase-Cas9 with sgRNA-HBc-1 and/or sgRNA-HBc2 was lentivirally transduced in HepG2.2.15.7 cells. Total DNA was collected at 5 days post-transduction, and mutations of HBV genome in HepG2.2.15.7 cells were detected by surveyor assay. The arrow indicated the surveyor digestion product. (**B**) HepG2.2.15.7 cells transduced with nickase-Cas9 and the sgRNAs were collected at 5 days post-transduction, and the expression of HBc was determined by immunoblotting. Intracellular HBV rcDNAs were extracted at 5 days post-transduction and quantified by qPCR. (**C**) Huh7 cells transduced with nickase-Cas9 and the sgRNAs were transfected with a plasmid containing 1.3-fold-overlength genome of HBV at 3 days post-transduction. Cell lysates were collected at 3 days post-transfection, and the expression of HBc was determined by immunoblotting. Intracellular HBV rcDNAs were extracted at 3 days post-transfection and quantified by qPCR. (**D**) HepG2-hNTCP-C4 cells expressing nickase-Cas9 and the sgRNAs by lentivirus vector were infected with HBV derived from the supernatants of HepAD38.7 cells at 3 days post-transduction. Cell lysates were collected at 10 days post-infection, and the expression of HBc protein was determined by immunoblotting. Intracellular HBV rcDNAs were extracted at 10 days post-infection and quantified by qPCR. *p < 0.01 vs. the results for control cells. (full-length gels are presented in Supplementary Figure).

To assess the effect of the expression of nickase-Cas9 together with one or both sgRNAs on the replication of HBV, nickase-Cas9 was lentivirally expressed with sgRNA-HBc-1 and/or sgRNA-HBc-2 in HepG2.2.15.7 cells. The surveyor assay revealed that mutations in the HBV genome were induced in HepG2.2.15.7 cells by the transduction of nickase-Cas9 together with both sgRNA-HBc-1 and sgRNA-HBc-2, but not in the cells transduced with either sgRNA singly (Fig. 3A). The expression of HBc protein and the production of rcDNA were suppressed in HepG2.2.15.7 cells upon the transduction of nickase-Cas9 with the pair of sgRNAs.

Interestingly, the transduction of nickase-Cas9 with either of the sgRNAs in HepG2.2.15.7 cells, which could not induce mutations in the HBV genome, suppressed the replication of HBV (Fig. 3B). In addition, the transduction of nickase-Cas9 with either of the sgRNAs in Huh7 cells transfected with 1.3-fold-overlength HBV-expressing plasmid and in HepG2-hNTCP-C4 cells infected with HBV suppressed the expression of HBc protein and the production of rcDNA (Fig. 3C,D). These results suggest that the expression of the nickase-Cas9 together with either or both of the sgRNAs can suppress the replication of HBV or through the formation of a complex that binds to the HBV genome without the requirement of DSBs on the targeted DNA.

### The binding of the Cas9 complex to the HBV genome suppressed HBV infection

**Figure 4:**
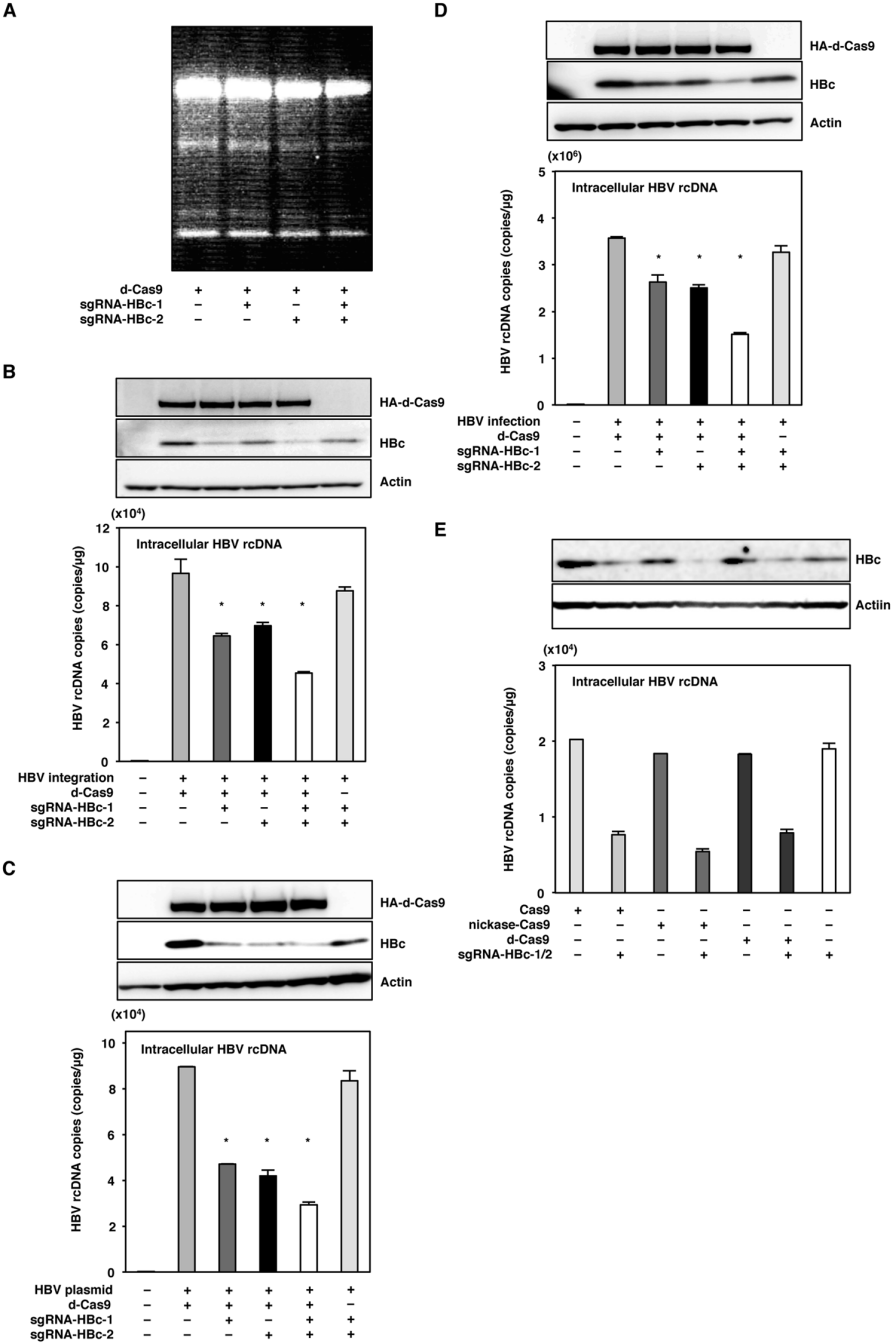
The binding of the Cas9 complex to the HBV genome suppressed HBV infection. (**A**) d-Cas9 with sgRNA-HBc-1 and/or sgRNA-HBc2 were lentivirally transduced in HepG2.2.15.7 cells. Total DNA was collected at 5 days post-transduction, and mutations of the HBV genome in HepG2.2.15.7 cells were detected by surveyor assay. (**B**) HepG2.2.15.7 cells transduced with d-Cas9 and the sgRNAs were collected at 5 days post-transduction, and the expression of HBc was determined by immunoblotting. Intracellular HBV rcDNAs were extracted at 5 days post-transduction and quantified by qPCR. (**C**) Huh7 cells transduced with d-Cas9 and the sgRNAs were transfected with a plasmid containing 1.3-fold-overlength genome of HBV at 3 days post-transduction. Cell lysates were collected at 3 days post-transfection, and the expression of HBc was determined by immunoblotting. Intracellular HBV rcDNAs were extracted at 3 days post-transfection and quantified by qPCR. (**D**) Nuclease dead Cas9 with the sgRNAs was lentivirally expressed in Huh7 cells. The plasmid containing 1.3-fold-overlength genome of HBV was transfected at 3 days post-transfection. Cell lysates were collected at 3 days post-transfection, and the intracellular HBc protein levels were determined by an immunoblotting analysis. Intracellular HBV rcDNAs were extracted at 3 days post-transfection and quantified by qPCR. (**D**) HepG2-hNTCP-C4 cells expressing d-Cas9 and the sgRNAs by lentivirus vector were infected with HBV derived from the supernatants of HepAD38.7 cells at 3 days post-transduction. Cell lysates were collected at 10 days post-infection, and the expression of HBc protein was determined by immunoblotting. Intracellular HBV rcDNAs were extracted at 10 days post-infection and quantified by qPCR. (**E**) Cas9, nickase-Cas9 and d-Cas9 with a pair of the sgRNAs were lentivirally expressed in HepG2.2.15.7 cells. The expression of HBc and the production of HBV rcDNAs were determined at 5 days post-transduction by immunoblotting and qPCR, respectively. *p < 0.01 vs. the results for control cells. (full-length gels are presented in Supplementary Figure).

To assess whether cleavage of the target genome is required in order to suppress the replication of HBV, we examined the effect of the expression of d-Cas9, which is unable to cleave but able to bind to the target DNA through formation of a complex with sgRNAs. The surveyor assay showed that no mutation in the HBV genome was induced in HepG2.2.15.7 cells by the transduction of d-Cas9 together with either or both of the sgRNAs (Fig. 4A). The expression of HBc protein and the production of rcDNA were suppressed in HepG2.2.15.7 cells upon the transduction of d-Cas9 with the sgRNAs (Fig. 4B). In addition, the transduction of d-Cas9 with the sgRNAs in Huh7 cells transfected with 1.3-fold-overlength HBV-expressing plasmid and in HepG2-hNTCP-C4 cells infected with HBV suppressed the expression of HBc protein and the production of rcDNA (Fig. 4C,D).

For a comparison of the suppressive effects of the expression of Cas9, nickase-Cas9 and d-Cas9 with the sgRNAs on the replication of HBV, these nucleases and sgRNAs were lentivirally transduced into HepG2.2.15.7 cells. The levels of suppression of HBc expression and rcDNA production in HepG2.2.15.7 cells by the expression of either Cas9, nickase-Cas9 or d-Cas9 together with the sgRNAs were comparable (Fig. 4E). These results suggest that the complex formation of the nucleases with sgRNAs binding to the target DNA is required for the suppression of HBV replication.

### Characterization of the Cas9-mediated suppression of HBV replication

To examine the mechanisms of the Cas9-mediated suppression of the replication of HBV, we conducted a northern blotting analysis to identify the viral RNAs including pgRNA and mRNAs in HepG2.2.15.7 cells transduced with Cas9, nickase-Cas9 and d-Cas9 together with the sgRNAs by lentivirus vectors. Although the production of HBV pgRNA was decreased by the expression of Cas9, nickase-Cas9 and d-Cas9 together with the sgRNAs, the production of PreS1 and PreS2/S mRNA was suppressed by the expression of Cas9 and nickase-Cas9 (but not by expression of d-Cas9) together with the sgRNAs.

**Figure 5:**
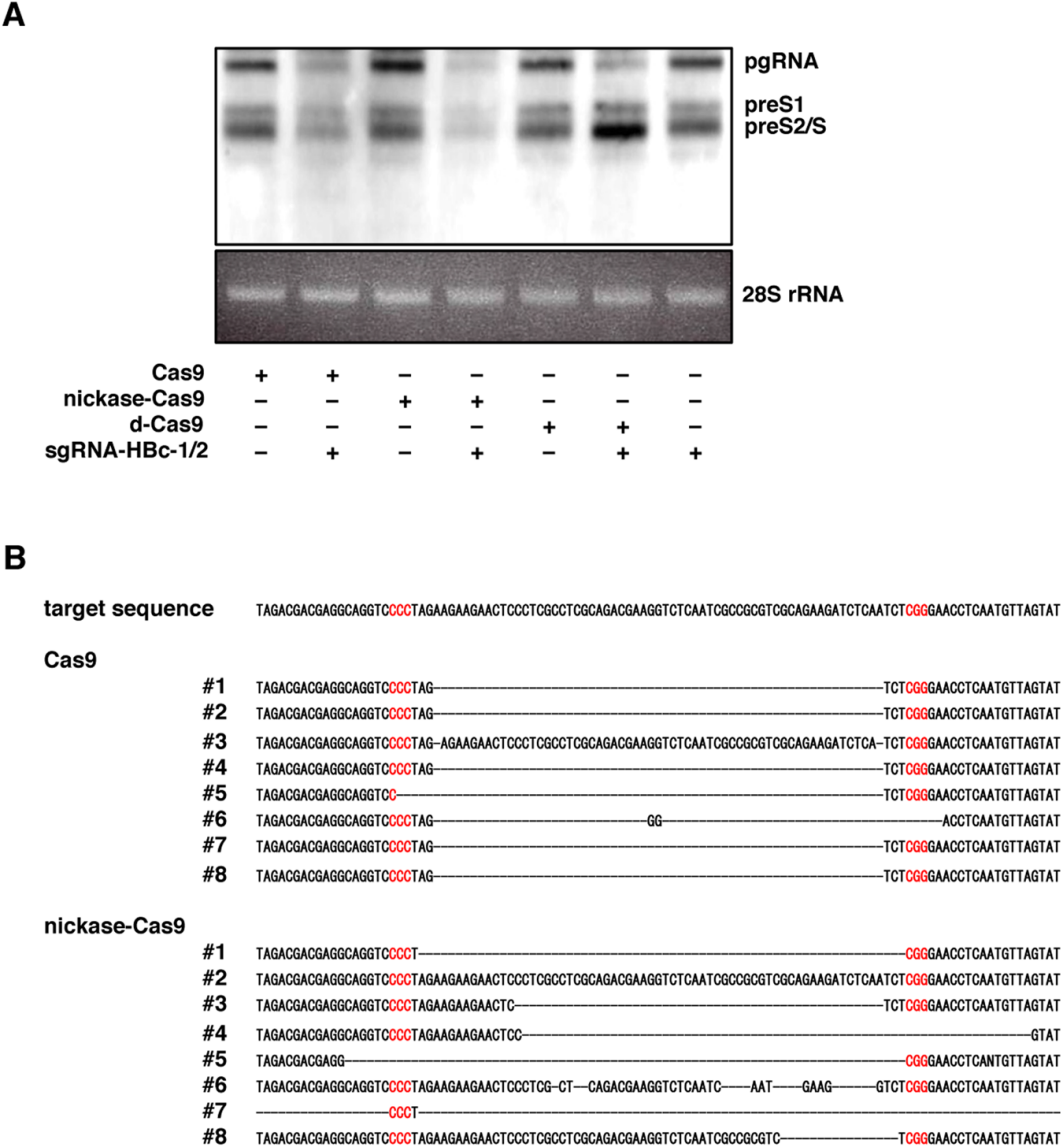
Characterization of the Cas9-mediated suppression of HBV replication. (**A**) HepG2.2.15.7 cells transduced with Cas9, nickase-Cas9 and d-Cas9 together with the pair of sgRNAs were collected at 5 days post-transduction, and the intracellular HBV RNA were determined by Northern blotting. (**B**) Total DNAs were extracted from HepG2.2.15.7 cells transduced with Cas9 and nickase-Cas9 together with the sgRNAs at 5 days post-transduction, and the target HBV sequences were directly sequenced after amplification. *p < 0.01 vs. the results for control cells. (full-length gels are presented in Supplementary Figure).

These results suggest that the expression of Cas9 and nickase-Cas9 with the sgRNAs induces mutation in the HBV genome, resulting in the suppression of whole HBV RNA transcription, whereas the expression of d-Cas9 with the sgRNAs was able to bind but unable to cleave the target HBV genome, resulting in the suppression of the transcription of pgRNA but not of the whole HBV RNA (Fig. 5A).

We next examined the mutation in the HBV genome induced by the expression of Cas9 or nickase-Cas9 together with the sgRNAs in HepG2.2.15.7 cells. In all eight clones, we detected deletions in HepG2.2.15.7 cells upon the expression of Cas9 with the sgRNAs, and most of the deletions were located at or immediately adjacent to the predicted Cas9 cleavage site. On the other hand, in seven of the eight clones, deletions of various lengths were detected in cells upon the expression of nickase-Cas9 with the sgRNAs (Fig. 5B).

The deep sequence analysis showed that the expression of Cas9 or nickase-Cas9 with the sgRNAs induced comparable mutations in HepG2.2.15.7 cells. As expected, expression of d-Cas9 with the sgRNAs did not induce any mutation (Table 2). These results suggest that the expression of Cas9, nickase-Cas9 and d-Cas9 together with the sgRNAs impairs the replication of HBV via the introduction of mutations into the HBV genome and the formation of a complex with the viral genome.

**Table 2:** Frequency of mutations induced by Cas9, nickase-Cas9 and d-Cas9 with a pair of sgRNAs.

Nuclease	Total read (count)	Indels mutations (count)	Mutation frequency (%)
Cas9	45210	25258	55.87
nickase-Cas9	44110	17210	39.02
d-Cas9	41938	86	0.21

### Cas9 and nickase-Cas9 with the sgRNAs cleaved the target HBV genome *in vivo*

**Figure 6:**
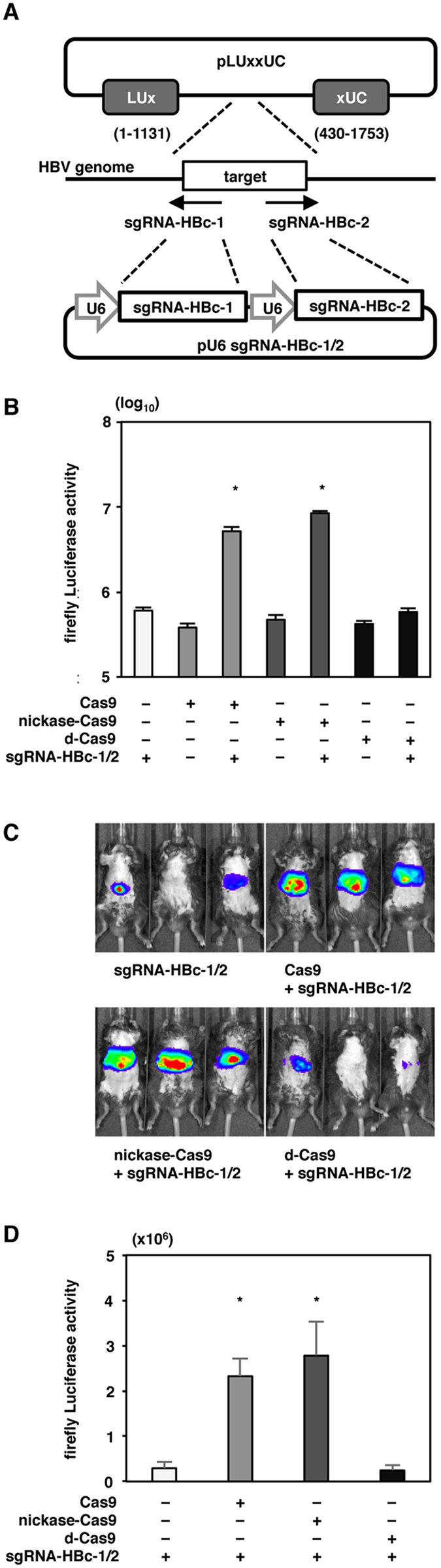
The effect of nickase-Cas9 with the pair of sgRNAs *in vivo*. (**A**) Schematic of the split LUxxUC plasmid inserted into the target HBc sequence and pU6-sgRNA-HBc-1/2 targeting the HBc sequence. A recombination of firefly luciferase is induced by DSBs on the inserted targeted sequence. (**B**) Cas9, nicakse-Cas9 and d-Cas9 with a pair of sgRNA-HBc-1/2-expressing plasmids were transfected with split LUxxUC plasmid into 293T cells. The firefly luciferase activity was determined at 24 hr post -transduction. (**C**,**D**) Cas9 and nickase-Cas9 plus the pair of sgRNA-HBc-1/2 or d-Cas9 plus the pair of sgRNA-HBc-1/2 with split LUxxUC-expressing plasmids were delivered into the livers of C57BL/6j mice via hydrodynamic injection (n = 3 mice per group). Luciferase activity was detected at 2 days post injection. The luciferase intensity was measured by an IVIS system. *p < 0.01 vs. the results for control cells.

Lastly, we examined whether the expression of nickase-Cas9 with both sgRNAs could cleave the target HBV genome *in vivo* by using a split LUxxUC plasmid in which the target HBV sequence was inserted. Recombination of the firefly luciferase gene was induced by DSBs on the inserted HBV genome sequence in the LUxxUC plasmid (Fig. 6A). Luciferase activity was increased by the expression of Cas9 and nickase-Cas9 but not by the expression of d-Cas9 using the pair of sgRNAs (Fig. 6B). We then introduced expression plasmids of the nucleases and the pair of sgRNAs into mice via hydrodynamic injection. High levels of luciferase expression were detected in the mice injected with Cas9, nickase-Cas9 and the pair of sgRNAs but not in those injected with d-Cas9 and the pair of sgRNAs (Fig. 6C,D), suggesting that cleavage of the targeted HBV genome is induced by the expression of Cas9 and nickase-Cas9 with both sgRNAs *in vivo*.

## Discussion

Removal of HBV cccDNA in nuclei, which serves as a template for all viral RNAs, including pgRNA and mRNAs for the expression of all viral proteins, is difficult with the current antiviral therapies. Thus, the elimination of cccDNA in nuclei is one of the major goals in the ongoing effort to cure HBV infection. Our present findings demonstrated that the expression of not only Cas9 but also that of nickase-Cas9 with sgRNAs can cleave the target HBV genome and suppress the replication of HBV. Our results also showed that the expression of d-Cas9 with the sgRNAs impairs the replication of HBV via the suppression of viral RNA transcription.

The CRISPR/Cas9 system was discovered in 2007 and shown to be a bacterial immune response system against phages^29^. It was subsequently found that Cas9 with CRISPR RNAs (crRNAs) and trans-activating crRNA (tracrRNA) can recognize and cleave the target DNAs^30^. An sgRNA which consists of a fusion of crRNA/tracrRNA was engineered, and the use of Cas9 with this sgRNA system was found to readily induce mutations in any targeted DNA sequence^14^. Several studies have already used the CRISPR/Cas9 system to target the HBV genome. Lin *et al*. first reported that Cas9 with sgRNAs targeting the HBV genome could reduce both the expressions of HBc and HBsAg in Huh7 cells transfected with an HBV expression plasmid and the expression of HBsAg in mice injected with an expression plasmid for HBV^6^.

In other reports, the expression of Cas9 with sgRNAs reduced the replication of HBV in HepAD38.7 cells^7^, HepG2-hNTCP-C4 cells infected with HBV^8^, and mice liver injected with the expression plasmid of HBV^9^. Karimova *et al*. reported that the expression of nickase-Cas9 with sgRNAs can suppress HBV replication *in vitro* ^31^. They showed that the expression of nickase-Cas9 with a pair of sgRNAs targeting the HBV genome encoding HBs and HBx induced mutations in the targeted genome and suppressed the expression of HBsAg. Another recent report demonstrated that nickase-Cas9 with multiple pairs of sgRNAs induced a large deletion in the HBV genome in HepG2 cells transfected with HBV expression plasmid^32^.

In the present study, we demonstrated that not only nickase-Cas9 but also d-Cas9 with sgRNAs targeting the HBV genome could suppress HBV infection. Previous study also showed that d-Cas9 with sgRNAs bound to target DNA sites and suppressed the gene expression^33, 34^. Northern blotting analysis revealed that d-Cas9 with the sgRNAs suppressed the expression of pgRNA which code core protein. So, the mechanism of suppressing effect of d-Cas9 with the sgRNAs on HBV replication was considered that silencing targeted mRNA expression resulted in suppression of HBV replication. The suppressive effect of HBV replication by d-Cas9, which lacked the ability to cleave the target HBV genome, was comparable to that achieved by Cas9 or nickase-Cas9. These results suggest that the suppressive effects of these nucleases on HBV replication are mediated mainly by the complex formation of nucleases with the sgRNAs binding to the target viral genomes, rather than by the cleavage and introduction of mutation in the HBV genome.

There have been several clinical trials using gene-editing techniques, with the first such study being conducted for treatment of patients infected with HIV. A gene encoding CCR5, one of the entry receptors for HIV, was knocked out of T cells derived from the patients by using zinc-finger nucleases, and the T cells were re-injected into the patients^10^. The second trial involved an infant with B-cell leukemia. Two genes involved in T-cell activity and survival, TCR and CD52, were knocked out of healthy donor-derived T cells by using transcription activator-like effector nucleases and injected into the patient^11^.

The first clinical trial using CRISPR/Cas9 was performed in patients in China who were resistant to the current therapy for metastatic lung cancers. The PD-1 gene in T cells derived from the patients was knocked out by CRISPR/Cas9 and re-injected into the patients^12^. Another clinical trial using CRISPR/Cas9 was performed in patients in the U.S. who were resistant to various therapies for cancers. Genes including NY-ESO-1, TCR and PD-1, which are involved in anti-cancer immunity, were modified in T cells from the patients by CRISPR/Cas9 and re-injected into the patients^13^. Since both clinical trials using CRISPR/Cas9 were applied *ex vivo*, the risk of undesirable off-target mutations in the host genome was limited.

However, for gene-editing therapies that target virus genes, like that considered in the present study, more careful attention should be paid to avoid undesirable off-target mutations in the host genome before advancing to *in vivo* applications in humans. Previous studies showed that various off-target mutations were induced by the CRISPR/Cas9 system^17–19^. The “paired nicking” strategy using nickase-Cas9 with a pair of sgRNAs was introduced to increase the specificity on the target sequences, and this approach achieved a 1500-fold reduction of off-target mutagenesis compared to the use of Cas9^20, 21^.

In conclusion, the expression of nickase-Cas9 as well as d-Cas9 together with a pair of sgRNAs suppressed the replication of HBV, suggesting that this strategy might be a promising therapeutic measure for chronic hepatitis B patients in whom a persistent presence of cccDNA is observed, and for integration of the HBV genome. However, further studies to validate the lack of off-target mutations are needed before clinical applications are considered.

## Methods

### Cell lines and viruses

All cell lines were cultured at 37 °C under the conditions of a humidified atmosphere and 5% CO_2_. The human hepatocellular carcinoma-derived Huh7 cells and human embryonic kidney-derived 293T cells were maintained in DMEM (Nacalai Tesque, Kyoto, Japan) supplemented with 100 U/ml penicillin, 100 μg/ml streptomycin (Sigma, St. Louis, MO), and 10% fetal bovine serum (FBS). The human hepatoblastoma-derived HepG2.2.15.7 cells and HepG2-hNTCP-C4 cells were maintained in the above medium supplemented with 400 μg/ml G418 (Nacalai Tesque). HepAD38.7 cells were cultured in DMEM/F-12 medium supplemented with 10% FBS, 100 U/ml penicillin, 100 μg/ml streptomycin, 18 μg/ml hydrocortisone (Sigma), 5 μg/ml insulin (Sigma), 400 μg/ml G418, and 400 ng/ml tetracycline (Nacalai Tesque). The HepG2.2.15.7 cells, HepG2-hNTCP-C4 cells and HepAD38.7 cells were kindly provided by Dr. T. Wakita.

The identification of human sodium taurocholate co-transporting polypeptide (hNTCP) as an HBV receptor led to generation of the HepG2-hNTCP-C4 cell line, which stably expresses hNTCP and therefore readily permits the infection and replication of HBV^27, 28^. For HBV infection, HepG2-hNTCP-C4 cells were seeded on 12-well plates (Iwaki, Tokyo, Japan) coated with collagen type 1 and incubated overnight. To obtain the HBV-containing culture supernatants, we cultured HepAD38.7 cells with tetracycline-free medium. Then, the HepG2-hNTCP-C4 cells were inoculated with 1,000 genome equivalents (GEq)/ml of HBV in the above medium supplemented with 2% DMSO (Sigma) containing 4% PEG 8000 (Nacalai Tesque), and the culture medium was replaced every 2 days.

### Plasmids

The plasmid pX330, which encodes hCas9 and sgRNA, was obtained from Addgene (Cambridge, MA; plasmid 44230). FLAG tag and NLS-appended hCas9 were amplified by polymerase chain reaction (PCR) and introduced into pCSII-EF, resulting in pCSII-EF-FLAG-Cas9. HA-tagged D10A mutation and D10A/H840A mutations of hCas9 were amplified by PCR and introduced into pCSII-EF, resulting in pCSII-EF-HA-nickase-Cas9 and pCSII-EF-HA-d-Cas9, respectively. The cassette of U6 sgRNA was introduced into pCSII-XhoI-HA-XbaI and designated pCSII-U6-sgRNA.

The fragments of guided RNA targeting the closed sites on both strands of HBV DNA encoding the HBc gene were inserted into the BsmB1 site of pCSII-U6-sgRNA and designated pCSII-U6-sgRNA-HBc-1 and pCSII-U6-sgRNA-HBc-2. The fragments of guided RNA targeting closed sites on both strands of HBV DNA encoding HBc were inserted into the Bbs1 site of pX330 and designated pX330-HBc-1 and pX330-HBc-2, respectively. cDNA of U6 sgRNA HBc-1 was inserted into the Age1 and Not1 sites of pX330-HBc-2 after deletion of hCas9c gene, and the resulting plasmid was designed pU6- sgRNA-HBc-1/2. The plasmid containing 1.3-fold-overlength genome of HBV genotype C, pUC19-C_JPNAT (accession no. AB246345), has been described previously^26^. The targeted sequence of the HBc gene was cloned into pCAG EgxxFP and pCAG LuxxUC. The plasmids used in this study were confirmed by direct sequencing with an ABI 3130 genetic analyzer (Thermo Fisher Scientific, Waltham, MA).

### Lipofection and lentiviral gene transduction

The lentiviral vectors and ViraPower Lentiviral Packaging Mix (Thermo Fisher Scientific) were co-transfected into 293T cells by Trans IT LT-1 (Mirus, Madison, WI), and the supernatants were recovered at 48 hr post-transfection. The lentivirus titer was determined by using a Lenti XTM qRT-PCR Titration Kit (Clontech, Mountain View, CA), and the expression levels and GFP were determined at 48 hr post-inoculation.

### Surveyor assay

The target HBc sequence was amplified from HepG2.2.15.7 cells using Takara Ex Taq DNA polymerase (Takara Bio, Shiga, Japan). The following primers were used for HBc sequences: 5′-ACCGTTATAGAGTA TTTGGT-3′ and 5′-GACAGGTACAGTAGAAGAAT-3′. Amplicons were purified after agarose gel electrophoresis using the Gel/PCR DNA Isolation System (Viogene, New Taipei City, Taiwan). The gel-purified PCR product was applied the Surveyor mutation detection kit (Integrated DNA Technologies, Tokyo, Japan) according to the manufacturer. Products were resolved by 10% gradient sodium dodecyl sulfate-polyacrylamide gel electrophoresis (SDS-PAGE) and bands were visualized by ethidium bromide staining and UV transilluminator.

### Immunoblotting

Cells were lysed on ice in lysis buffer (20 mM Tris-HCl [pH 7.4], 135 mM NaCl, 1% Triton-X 100, 10% glycerol) supplemented with a protease inhibitor mix (Nacalai Tesque), boiled in loading buffer and subjected to 5–20% gradient sodium dodecyl sulfate-polyacrylamide gel electrophoresis (SDS-PAGE). The proteins were transferred to polyvinylidene difluoride membranes (Millipore, Bedford, MA) and reacted with the appropriate antibodies. The immune complexes were visualized with SuperSignal West Femto Substrate (Pierce, Rockford, IL) and detected by using an LAS-3000 image analyzer system (Fujifilm, Tokyo).

### Antibodies

Mouse monoclonal antibody to β-actin and horseradish peroxidase (HRP)-conjugated mouse monoclonal antibody to FLAG were purchased from Sigma. Rat anti-HA antibody was purchased from Roche Diagnostics (Indianapolis, IN). Mouse anti-HBc antibody was kindly provided by Dr. A. Ryo. Six-diamidono-2-phenylindole (DAPI) was purchased from Vector Laboratories (Burlingame, CA).

### Immunofluorescence assay

Cells cultured on glass slides were fixed with 4% paraformaldehyde (PFA) in phosphate-buffered saline (PBS) at room temperature for 30 min. Cell nuclei were stained with DAPI. Cells were observed with a FluoView FV1000 laser scanning confocal microscope (Olympus, Tokyo).

### Northern blotting

Total RNA (2 μg) was subjected to electrophoresis in 1.2% agarose-formaldehyde gel and morpholinepropanesulfonic acid (MOPS) buffer. Ribosomal RNA was visualized by ethidium bromide staining, and electrophoresed RNA was transferred onto a positively charged nylon membrane (Roche). An RNA probe was synthesized by a digoxigenin (DIG) RNA labeling kit (Roche). Hybridization and detection were performed by using a DIG Northern Starter kit (Roche) according to the manufacturer’s protocols. The signals were detected by an LAS-3000 image analyzer system (Fujifilm).

### Purification of intracellular and extracellular HBV rcDNA and cellular RNA

Extracellular and intracellular HBV DNA were extracted as described previously^33^. Briefly, the cell pellets were lysed by lysis buffer (50 mM Tris-HCl [pH 7.4], 1 mM EDTA, 1% NP-40) at 4 °C for 15 min. After centrifugation at 15,000 rpm for 5 min, the supernatant was incubated with 7 mM magnesium acetate (MgOAc), 0.2 mg/ml of Dnase I (Roche), and 0.1 mg/ml of Rnase A (Sigma) at 37 °C for 3 hr. After the addition of 10 mM EDTA, the lysates were digested by proteinase K (0.3 mg/ml; Thermo Fisher Scientific) and 2% SDS at 37 °C for 12 hr. Extracted HBV DNA was purified by phenol-chloroform-isoamyl alcohol, precipitated with ethanol, and resolved in pure water. Total RNA was extracted by using a Pure Link RNA Mini Kit (Thermo Fisher Scientific) according to the manufacturer’s protocol.

### qPCR

Quantitative PCR (qPCR) for HBV rcDNAs was performed by using Fast SYBR green master mix (Applied Biosystems, Foster City, CA). The following primers were used for the detection of HBV DNA: 5′-GGAGGGATACATAGAG GTTCCTTGA-3′ and 5′-GTTGCCCGTTTGTCCTCTAATTC-3′.

### Direct sequencing

The target HBc sequence was amplified from HepG2.2.15.7 cells using PrimeSTAR GXL DNA polymerase (Takara Bio, Shiga, Japan). The following primers were used for HBc sequences: 5′-ACCGTTATAGAGTA TTTGGT-3′ and 5′-GACAGGTACAGTAGAAGAAT-3′. Amplicons were purified after agarose gel electrophoresis using the Gel/PCR DNA Isolation System (Viogene, New Taipei City, Taiwan). The gel-purified PCR product was cloned with the use of a Zero Blunt TOPO PCR Cloning Kit (Thermo Fisher Scientific) according to the manufacturer’s instructions. The target HBc sequences were determined by direct sequencing using the BigDye Terminator v3.1 Cycle Sequencing Kit and an ABI3130 Genetic Analyzer (Applied Biosystems).

### Deep sequencing

The possible off-target and HBc sequences were amplified from HepG2.2.15.7 cells and adapters for next-generation sequencing. The primers used for the detection of the sequences of the possible off-target sites and HBV genome are given in Table 3. A PCR using 250-ng template DNA was performed under the following conditions: 35 cycles of 98 °C for 10 sec, 55 °C for 15 sec and 68 °C for 30 sec using PrimeSTAR GXL DNA polymerase (Takara Bio). Amplicons were purified after agarose gel electrophoresis using the Gel/PCR DNA Isolation System (Viogene). Paired-end sequencing was performed with 300 cycles on a MiSeq sequencer (Illumina, San Diego, CA).

**Table 3:** The primers used for the detection of the sequences of the possible off-target sites and HBV genome.

**Off-target #1–1**:
5′-TCGTCGGCAGCGTCAGATGTGTATAAGAGACAGTTTGTCCCTTTGACTTGTAGTCC-3′
and
5′-GTCTCGTGGGCTCGGAGATGTGTATAAGAGACAGGGAATGGGAAGGCATTTACG-3′
**Off-target #1–2**:
5′-TCGTCGGCAGCGTCAGATGTGTATAAGAGACAGGAAACTAAGAGAGAAACGGCATG-3′
and
5′-GTCTCGTGGGCTCGGAGATGTGTATAAGAGACAGAAATGTTTCAAGATGGGCCTC-3′
**Off-target #1–3**:
5′-TCGTCGGCAGCGTCAGATGTGTATAAGAGACAGCCAACCAATGGATCAGGACG-3′
and
5′-GTCTCGTGGGCTCGGAGATGTGTATAAGAGACAGCCTCAAAGAAAGGAACAACAGC-3′
**Off-target #2–1**:
5′-TCGTCGGCAGCGTCAGATGTGTATAAGAGACAGACAGGCATAAGACACCATACC-3′
and
5′-GTCTCGTGGGCTCGGAGATGTGTATAAGAGACAGCAAAGACCGGGTTCCAGATG-3′
**Off-target #2–2**:
5′-TCGTCGGCAGCGTCAGATGTGTATAAGAGACAGTCTGGGAAGAATAAGGGTATTGG-3′
and
5′-GTCTCGTGGGCTCGGAGATGTGTATAAGAGACAGTGGAGTCCACACCTGTTG-3′
**Off-target #2–3**:
5′-TCGTCGGCAGCGTCAGATGTGTATAAGAGACAGAAGTGGGTCCTCAGTGAAAC-3′
and
5′-GTCTCGTGGGCTCGGAGATGTGTATAAGAGACAGTACCTTGCCTTCCTTCATGG-3′
**HBc**:
5′-TCGTCGGCAGCGTCAGATGTGTATAAGAGACAGACCGTTATAGAGTATTTGGT-3′
and
5′-GTCTCGTGGGCTCGGAGATGTGTATAAGAGACAGGACAGGTACAGTAGAAGAAT-3′

### Reporter assay

pCSII-EF-Cas9 or pCSII-EF-nickase-Cas9 with or without pU6-sgRNA-HBc-1/2 together with pCAG LuxxUC were transfected into 293T cells and luciferase activity in the cell lysates was determined with the use of the Luciferase Assay System (Promega, Madison, WI) according to the manufacturer’s protocol at 24 hr post-transfection.

### Animal studies

We injected C57BL/6j mice with a mixture of 3.3 μg Cas9, nickase-Cas9 or d-Cas9 and 3.3 μg U6-sgRNA-HBc-1/2 together with 3.3 μg pCAG LUxxUC by the hydrodynamic injection technique. Plasmids were dissolved in 1.8 ml of saline, and the mixture was injected through the tail vein over a 10-sec period. At 48 hr post-injection, the mice were examined with IVIS Spectrum *In Vivo* Imaging System (PerkinElmer, San Jose, CA). All animal experiments conformed to the Guidelines for the Care and Use of Laboratory Animals and were approved by the Institutional Committee of Laboratory Animal Experimentation (Research Institute for Microbial Diseases, Osaka University).

### Statistics

The results are expressed as the means ± standard deviation. The significance of differences in means was determined by Student’s *t*-test.

## Electronic supplementary material

Supplementary Information

## References

[1] Dienstag JL (2008). Hepatitis B virus infection. N. Engl. J. Med..

[2] Ganem D, Varmus HE (1987). The molecular biology of the hepatitis B viruses. Annu. Rev. Biochem..

[3] Lucifora J (2014). Specific and nonhepatotoxic degradation of nuclear hepatitis B virus cccDNA. Science.

[4] Zoulim F (2011). Hepatitis B virus resistance to antiviral drugs: where are we going?. Liver Int..

[5] Wursthorn K (2006). Peginterferon alpha‐2b plus adefovir induce strong cccDNA decline and HBsAg reduction in patients with chronic hepatitis B. Hepatology.

[6] Lin SR (2014). The CRISPR/Cas9 system facilitates clearance of the intrahepatic HBV templates *in vivo*. Mol. Ther. Nucleic Acids.

[7] Kennedy EM (2015). Suppression of hepatitis B virus DNA accumulation in chronically infected cells using a bacterial CRISPR/Cas RNA-guided DNA endonuclease. Virology.

[8] Seeger C, Sohn JA (2014). Targeting hepatitis B virus with CRISPR/Cas9. Mol. Ther. Nucleic Acids.

[9] Ramanan, V., Shlomai, A., Cox, D. B. T. & Schwartz, R. E. CRISPR/Cas9 cleavage of viral DNA efficiently suppresses hepatitis B virus. *Sci*. *Rep*., doi:10.1038/srep10833 (2015). 10.1038/srep10833 PMC4649911 26035283

[10] Tebas P (2014). Gene editing of CCR5 in autologous CD4 T cells of persons infected with HIV. N. Engl. J. Med..

[11] Qasim W (2015). First clinical application of talen engineered universal CAR19 T cells in B-ALL. Blood.

[12] Cyranoski D (2016). Chinese scientists to pioneer first human CRISPR trial. Nature.

[13] Reardon, S. First CRISPR clinical trial gets green light from US panel. *Nature*, doi:10.1038/nature.2016.20137 (2016).

[14] Jinek M (2012). A programmable dual-RNA-guided DNA endonuclease in adaptive bacterial immunity. Science.

[15] Le C (2013). Multiplex genome engineering using CRISPR/Cas systems. Science.

[16] Mali P (2013). RNA-guided human genome engineering via Cas9. Science.

[17] Fu Y (2013). High-frequency off-target mutagenesis induced by CRISPR-Cas nucleases in human cells. Nat. Biotechnol..

[18] Pattanayak V (2013). High-throughput profiling of off-target DNA cleavage reveals RNA-programmed Cas9 nuclease specificity. Nat. Biotechnol..

[19] Hsu PD (2013). DNA targeting specificity of RNA-guided Cas9 nucleases. Nat. Biotechnol..

[20] Ran FA (2013). Double nicking by RNA-guided CRISPR Cas9 for enhanced genome editing specificity. Cell.

[21] Bin S (2014). Efficient genome modification by CRISPR-Cas9 nickase with minimal off-target effects. Nat. Methods.

[22] Okamoto H (1988). Typing hepatitis B virus by homology in nucleotide sequence: comparison of surface antigen subtypes. J. Gen. Virol..

[23] Mashiko D (2013). Generation of mutant mice by pronuclear injection of circular plasmid expressing Cas9 and single guided RNA. Sci. Rep..

[24] Naito, Y., Hino, K., Bono, H. & Ui-Tei, K. CRISPRdirect: software for designing CRISPR/Cas guide RNA with reduced off-target sites. *Bioinformatics*, doi:10.1093/bioinformatics/btu743 (2014). 10.1093/bioinformatics/btu743 PMC4382898 25414360

[25] Sells MA, Mei-Lin C, Acs G (1987). Production of hepatitis B virus particles in Hep G2 cells transfected with cloned hepatitis B virus DNA. Proc. Natl. Acad. Sci. USA.

[26] Sugiyama M (2006). Influence of hepatitis B virus genotypes on the intra- and extracellular expression of viral DNA and antigens. Hepatology.

[27] Yan H (2012). Sodium taurocholate cotransporting polypeptide is a functional receptor for human hepatitis B and D virus. eLife.

[28] Watashi K (2014). Cyclosporin A and its analogs inhibit hepatitis B virus entry into cultured hepatocytes through targeting a membrane transporter, sodium taurocholate cotransporting polypeptide (NTCP). Hepatology.

[29] Barrangou R (2007). CRISPR provides acquired resistance against viruses in prokaryotes. Science.

[30] Deltcheva E (2011). CRISPR RNA maturation by trans-encoded small RNA and host factor RNase III. Nature.

[31] Karimova M (2015). CRISPR/Cas9 nickase-mediated disruption of hepatitis B virus open reading frame S and X. Sci Rep.

[32] Sakuma T (2016). Highly multiplexed CRISPR-Cas9-nuclease and Cas9-nickase vectors for inactivation of hepatitis B virus. Genes Cells.

[33] Qi LS (2013). Repurposing CRISPR as an RNA-guided platform for sequence-specific control of gene expression. Cell.

[34] Sander JD, Joung JK (2014). CRISPR-Cas systems for editing, regulating and targeting genomes. Nat Biotechnol..

