# Supplementary material for: Suppression of HBV replication by the expression of nickase- and nuclease dead-Cas9
Source: Sci Rep. 2017 Jul 21;7:6122. doi: 10.1038/s41598-017-05905-w (PMC5522428; doi:10.1038/s41598-017-05905-w)
Supplement: Supplementary file 1 — Supplementary Information [file 41598_2017_5905_MOESM1_ESM.pdf]

Suppression of HBV replication by the expression of nicase- and nuclease dead Cas9

Takeshi Kurihara, Takasuke Fukuhara, Chikako Ono, Satomi Yamamoto, Kentaro Uemura, Toru Okamoto, Masaya Sugiyama, Daisuke Motooka, Shota Nakamura, Masato Ikawa, Masashi Mizokami, Yoshihiko Maehara, and Yoshiharu Matsuura.

full-length blots for Fig. 2A, 3A, 4A

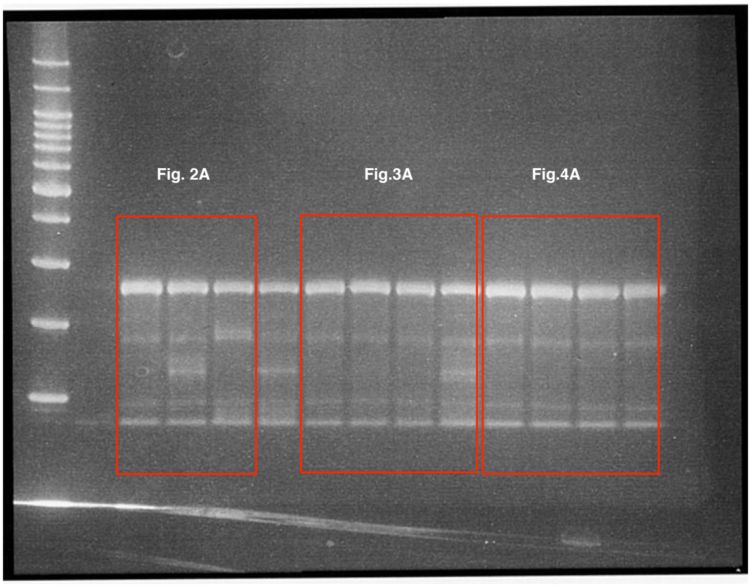

Supplementary Figure
The full-length blots for Figure 2A, 3A, 4A
Dashed line boxes indicate the cropped images used in Fig. 2A, 3A, 4A.

full-length blots for Fig. 2B

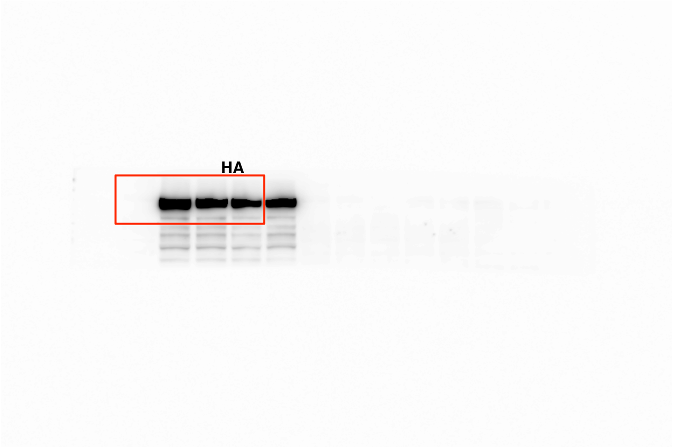

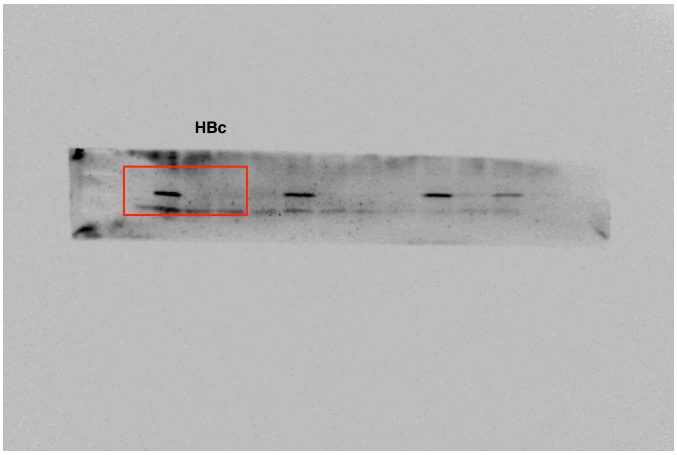

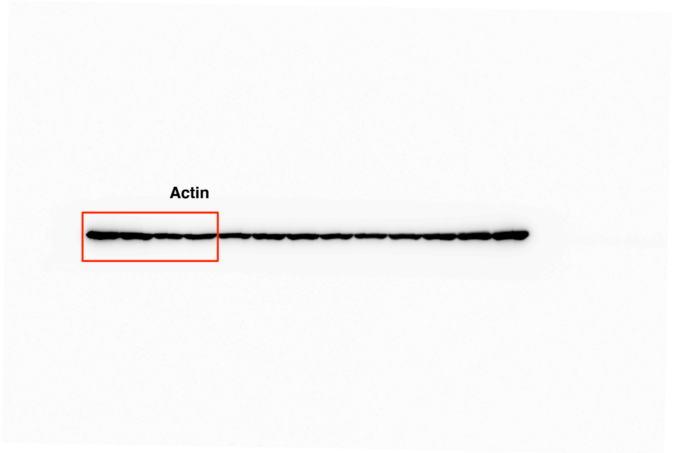

full-length blots for Fig. 2C

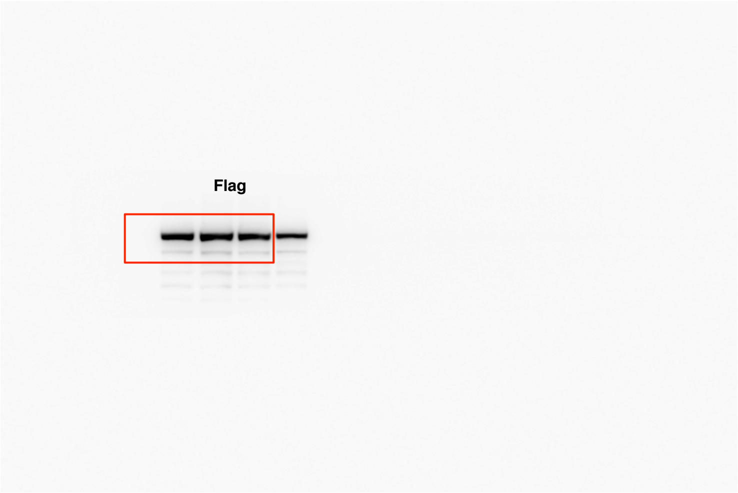

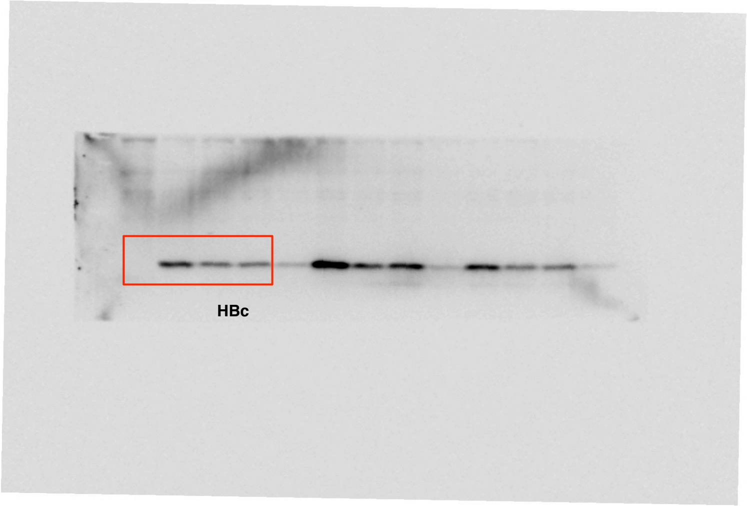

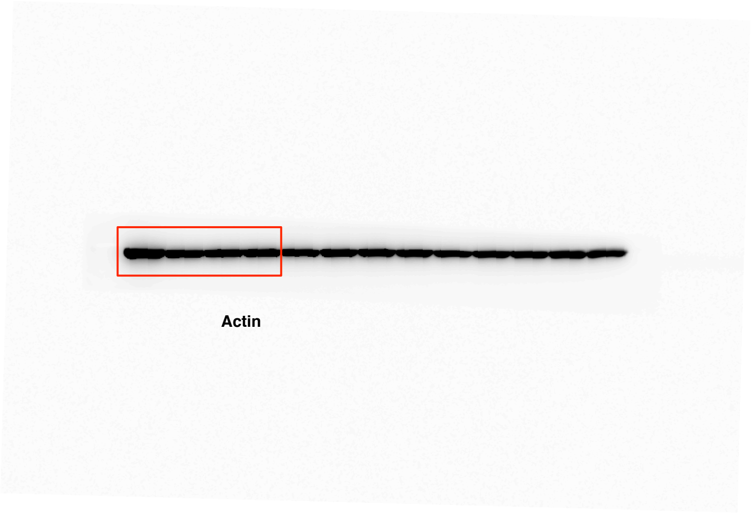

full-length blots for. Fig. 2D

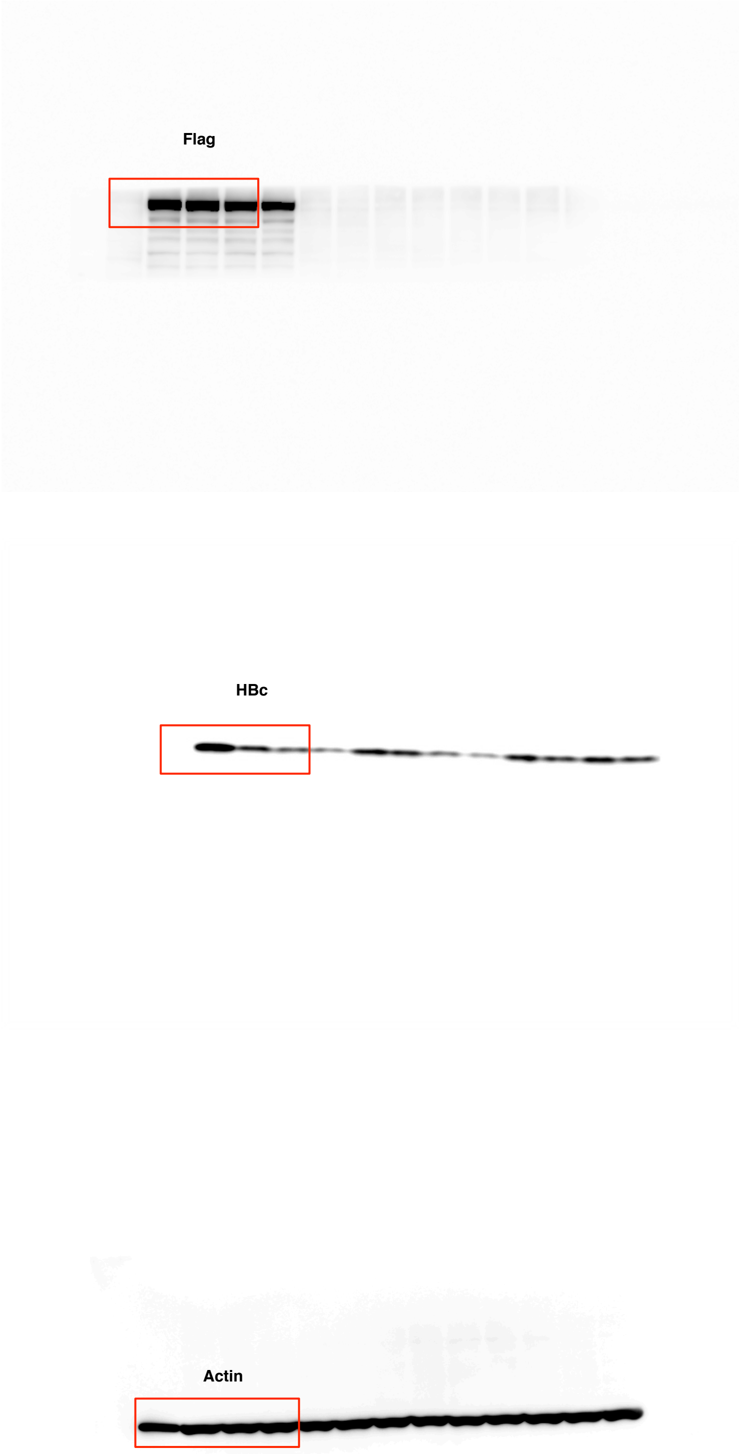

full-length blots for Fig. 3B

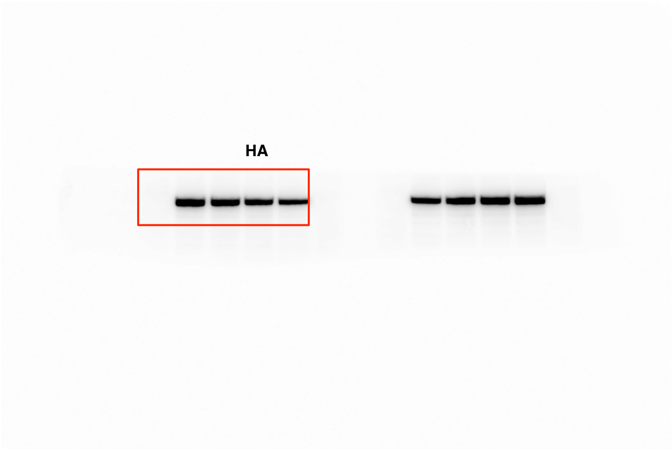

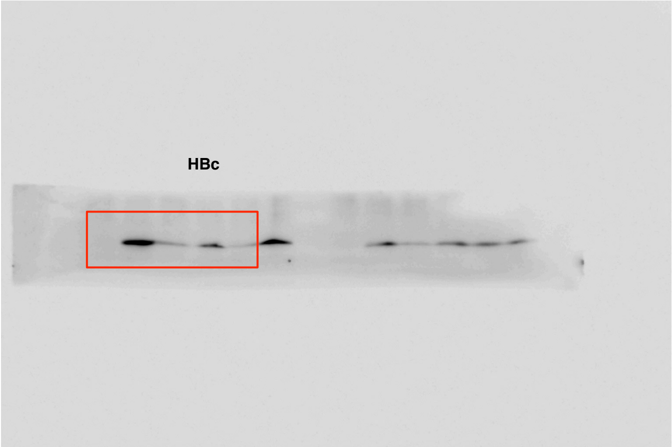

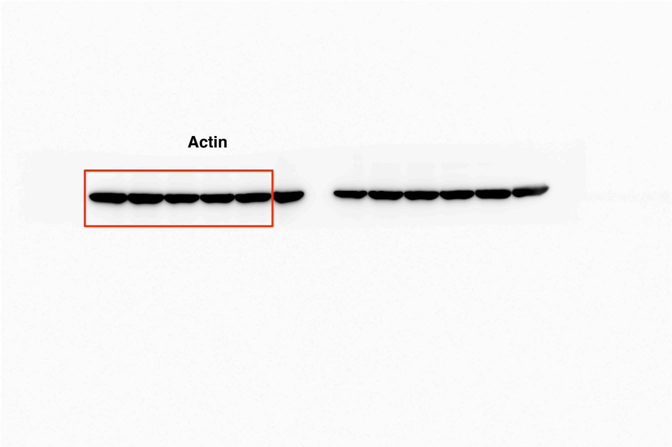

full-length blots for 3C

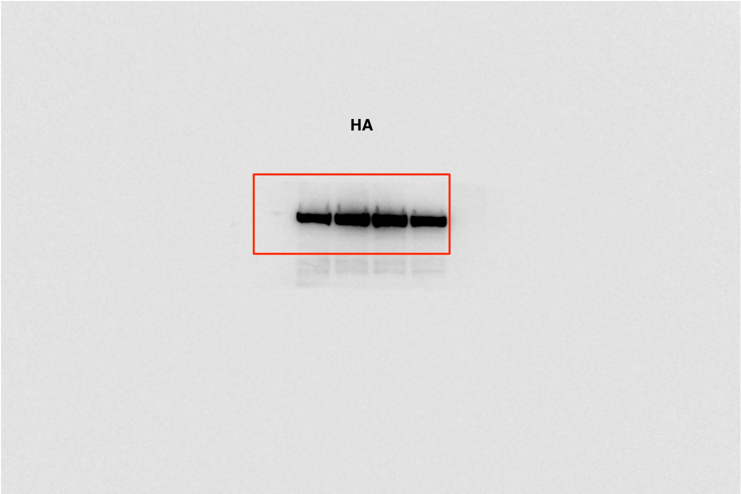

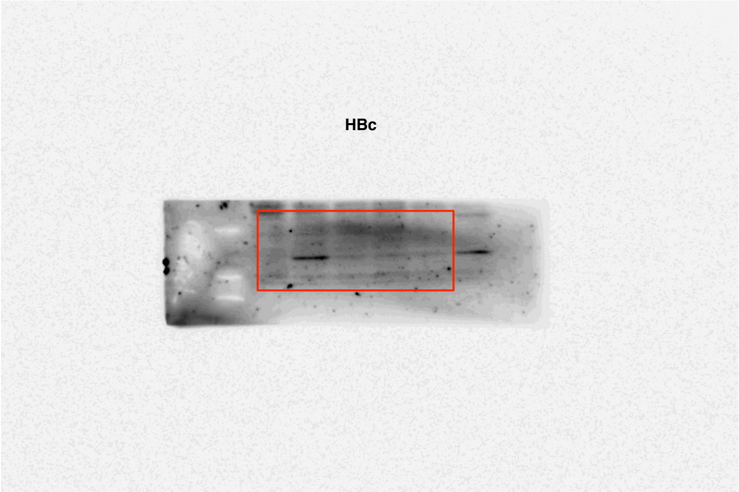

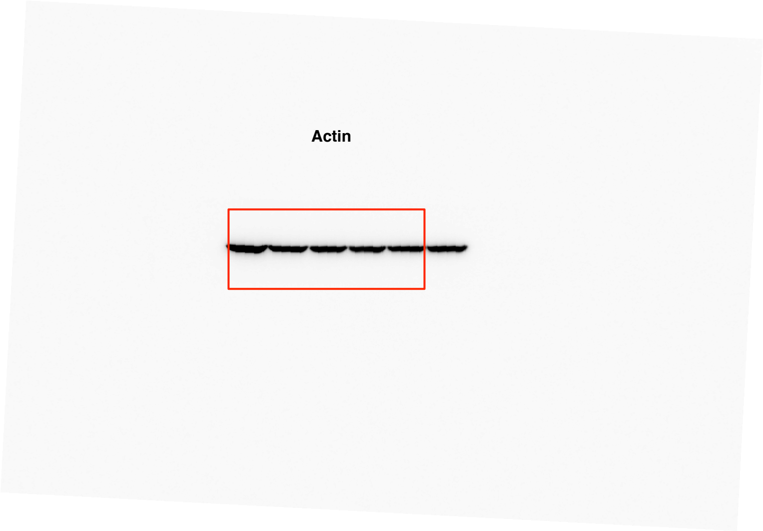

full-length blots for Fig. 3D

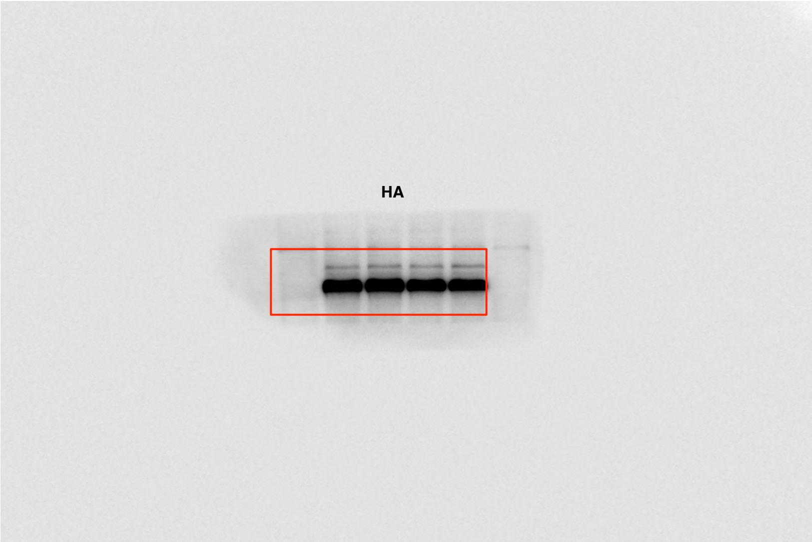

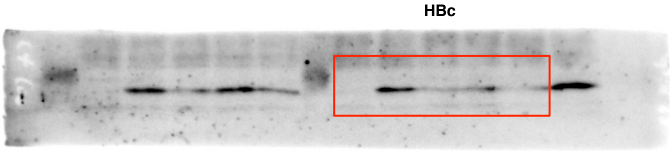

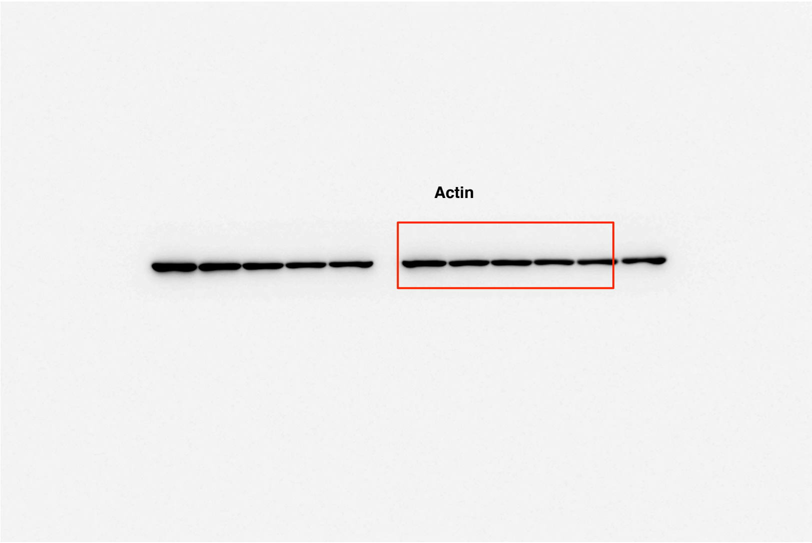

full-length blots for Fig. 4B

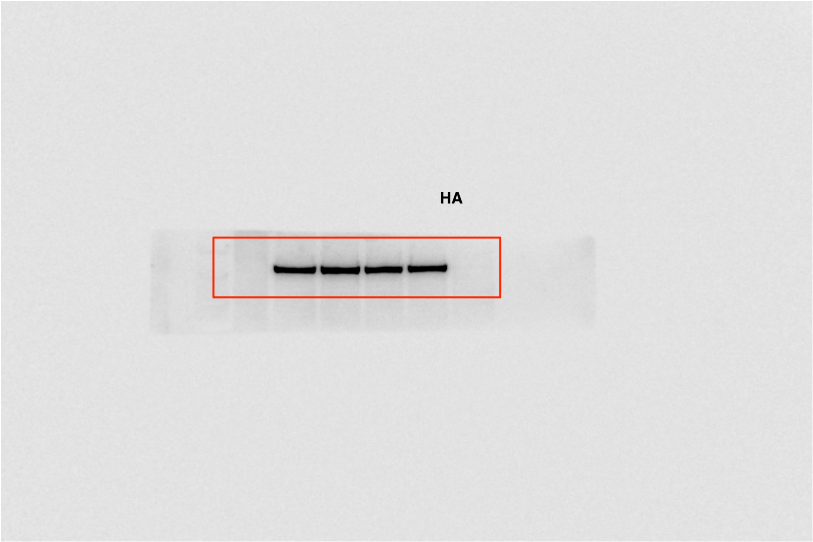

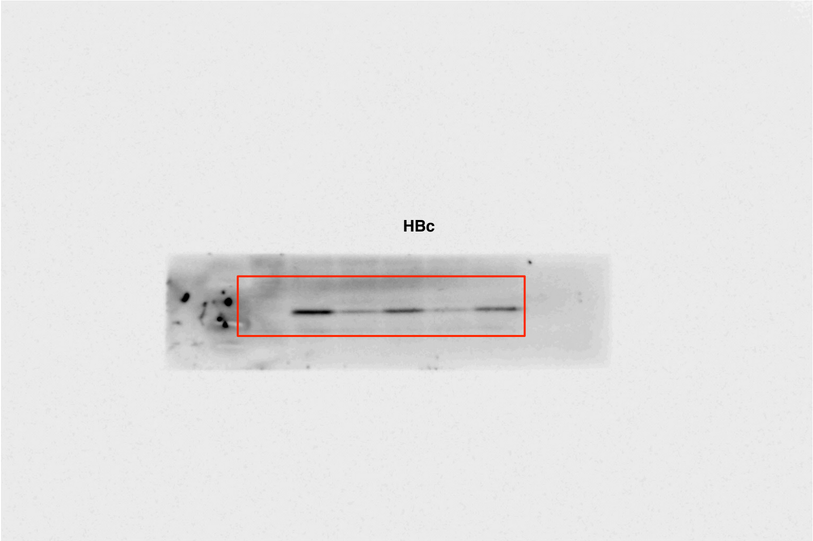

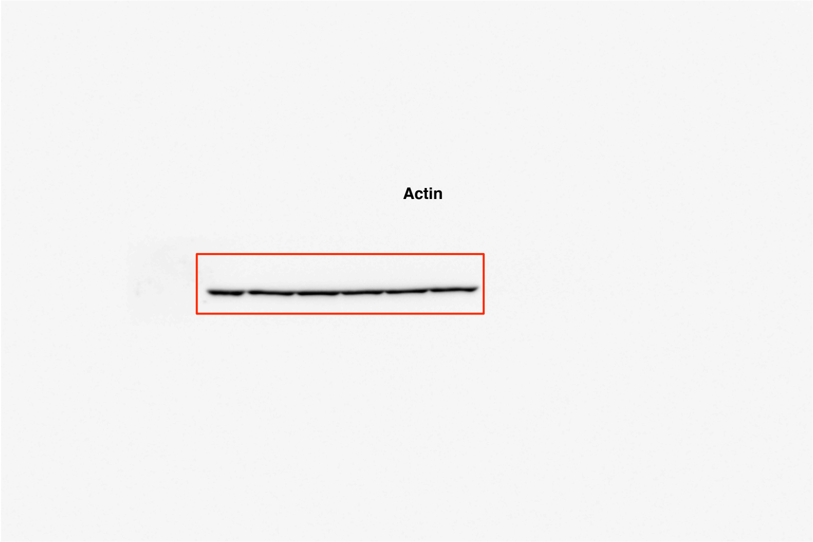

full-length blots for Fig. 4C

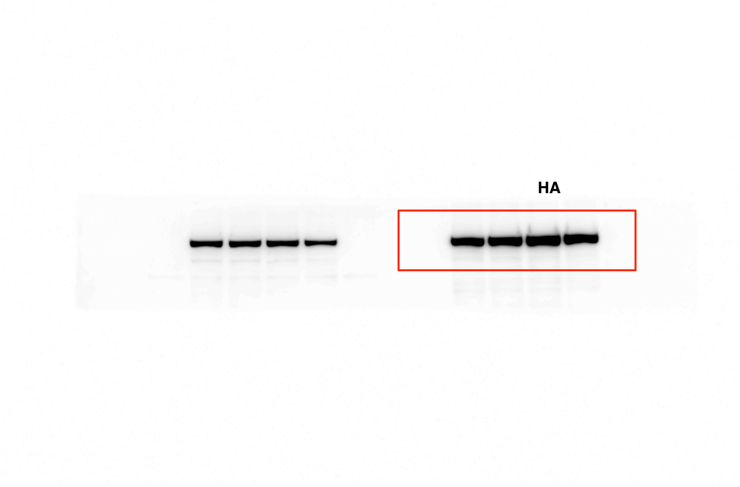

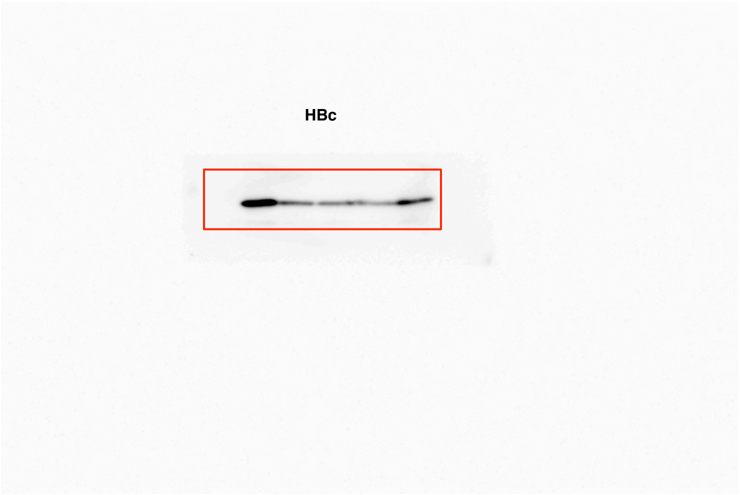

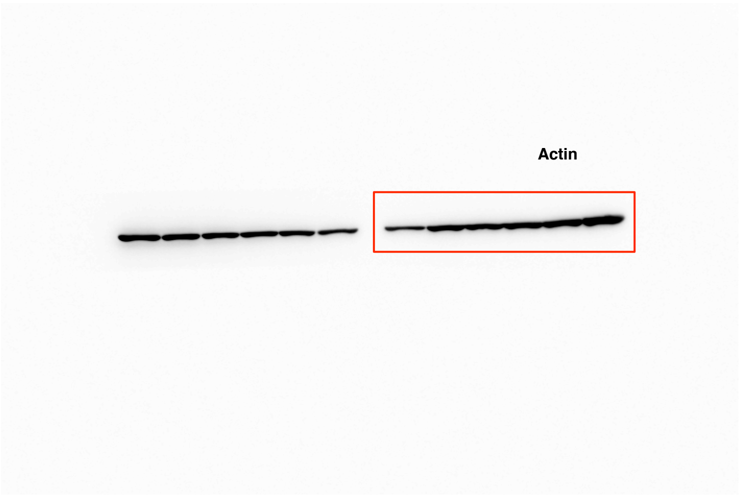

full-length blots for Fig. 4D

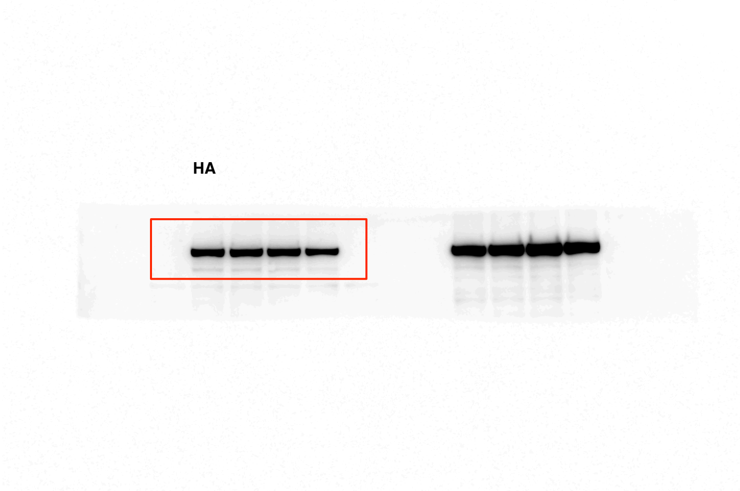

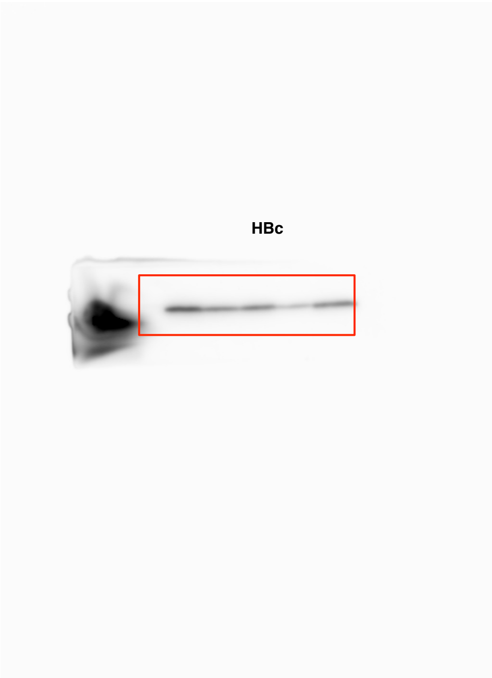

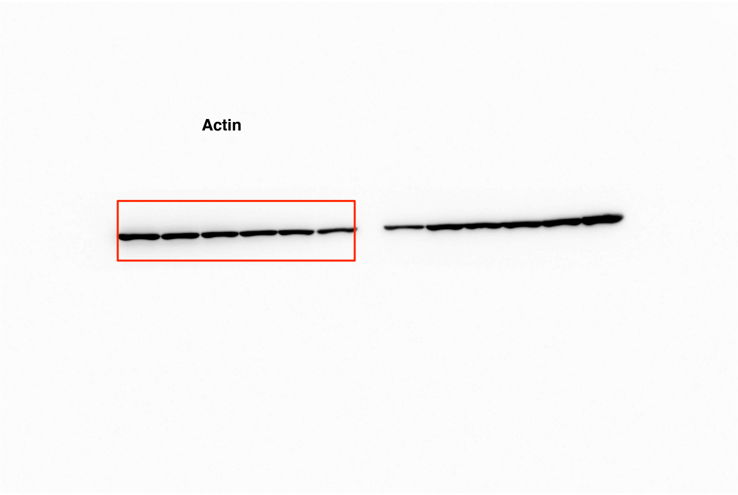

full-length blots for Fig. 4E

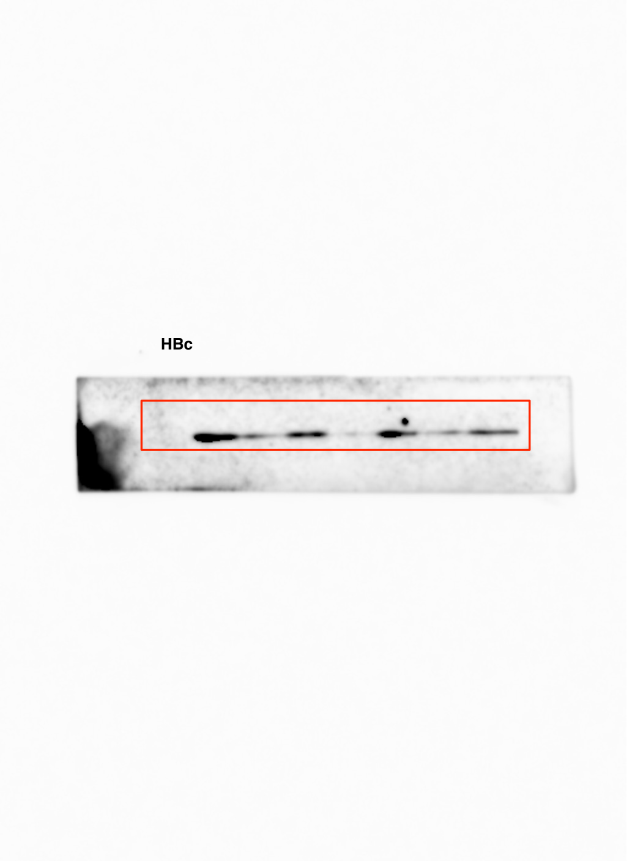

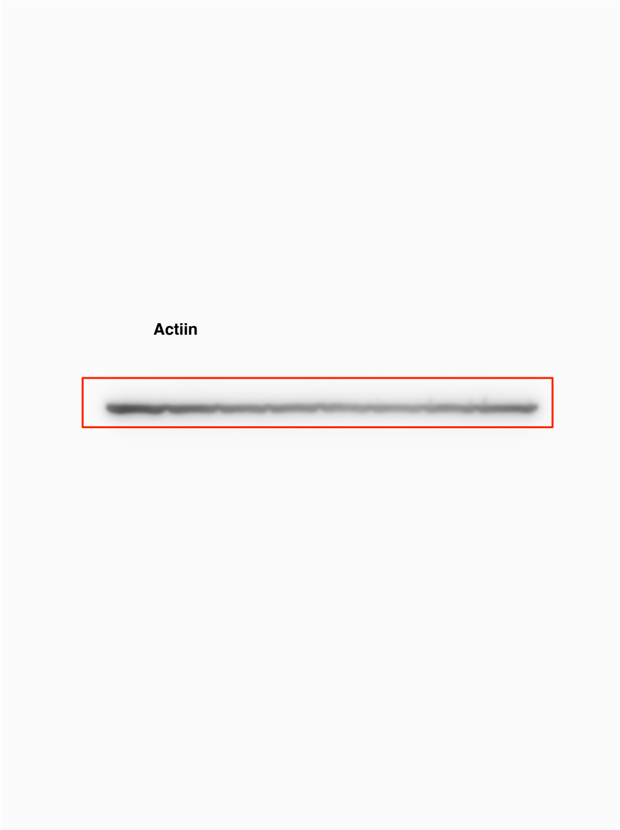

full-length blots for Fig. 5A

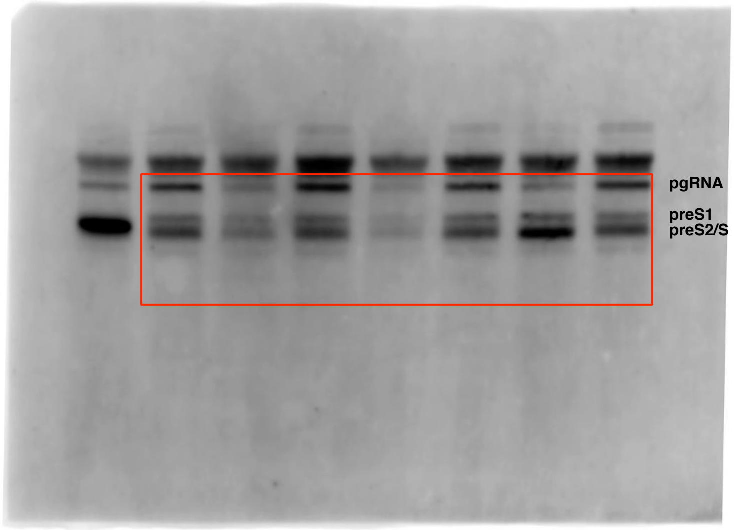

Ribosomal RNA

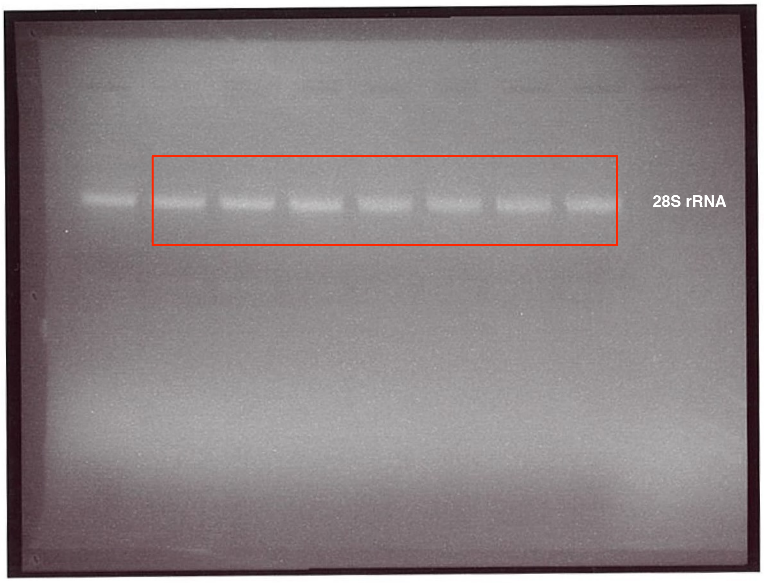

GFP Cas9 Cas9 sgRNA Nick Nick sgRNA dCas dCas sgRNA sgRNA
